# Health-related quality of life and influencing factors in parents of children with congenital heart disease: a systematic review and meta-analysis

**DOI:** 10.3389/fpubh.2025.1622491

**Published:** 2025-10-10

**Authors:** Yu Wang, Fang Ma, Jingran Yang, Yimei Zhang, Min Zhou, Yangjuan Bai, Majd Elmobasher, Zhisong Chen

**Affiliations:** ^1^School of Nursing, Kunming Medical University, Kunming, China; ^2^Department of Nursing, The First Affiliated Hospital of Kunming Medical University, Kunming, China; ^3^Cardiology Department, The First Affiliated Hospital of Kunming Medical University, Kunming, China; ^4^Cardiology Department II, The First Affiliated Hospital of Kunming Medical University, Kunming, China

**Keywords:** congenital heart disease, health-related quality of life, parents, meta-analysis, systematic review

## Abstract

**Introduction:**

Parents of children with congenital heart disease (CHD) have been described as ‘hidden patients,’ which negatively impacts their health-related quality of life (HRQoL). The extent of HRQoL impairment among parents of children with CHD and its contributing factors remain controversial. This systematic review and meta-analysis aimed to systematically examine the HRQoL in parents of children with CHD and sought to determine the influencing factors.

**Methods:**

A comprehensive search for articles was performed via CINAHL, Embase, PsycINFO, PubMed, Web of Science, China National Knowledge Infrastructure (CNKI), Chinese Biomedical (Sinomed), Weipu (VIP), and Wanfang databases from the establishment of the database to September 30, 2024. Cross-sectional, cohort and case–control studies, evaluating the HRQoL or influencing factors for parents of children with CHD were identified and collected.

**Results:**

Of the 4,013 studies identified, one cohort study and 22 cross-sectional studies comprising 3,681 parents of children with CHD were included. Pooled results indicated that in each domain of the Short Form-36 Health Survey (SF-36), the HRQoL scores for general health (SMD, −0.58; 95% CI, −0.79, −0.37; *p* < 0.001), role difficulty due to emotional problems (SMD, −0.79; 95% CI, −1.00, −0.58; *p* < 0.001), role difficulty due to physical problems (SMD, −0.31; 95% CI, −0.52, −0.11; *p* = 0.003), and social function domains (SMD, −0.53; 95% CI, −0.74, −0.33; *p* < 0.001) in mothers of children with CHD were lower than those in mothers of healthy children. There was a negative correlation between negative coping and HRQoL in parents of children with CHD (*r* = −0.07, *p* = 0.003).

**Conclusion:**

Compared with the general population, mothers of children with CHD have lower social function and general health and more role difficulty due to emotional and physical problems. Meanwhile, negative coping is a potential influencing factor in the HRQoL of parents of children with CHD. Notably, SF-36 scores did not significantly differ in mental health, physical function, vitality, and pain domains between mothers of children with CHD and the general population, from our result was that, regardless of the mental or physical health scores, and even in the total HRQoL scores, there were no statistically significant differences between fathers and mothers who had a CHD child.

## Introduction

Relatively high prevalence, significant morbidity and mortality of congenital heart disease (CHD), is the leading cause of death from non-communicable diseases (NCDs) in those under 20 years of age, which has become the global burden that international health policy needs to focus on (1, 2). Since 1995, almost 9 out of 10 CHD children can reach adulthood, and most of them require surgery, hospitalizations, and long-term home care (3, 4). Therefore, parents have to take on many complex care-related tasks, such as symptom management and medication therapy (5). Parents caring for children with CHD, require necessitate substantial effort both during the day and night. Simultaneously, having a child diagnosed with CHD means that the parents has to face costly treatment, travel for care and a reduction income, which lead to financial hardship (6–8). All of the above result in patients’ sleep disruption and chronic sleep deprivation (4, 9), and negatively impact parent health. Parents of children with CHD have been described as ‘hidden patients,’ with a high prevalence of psychological distress (10), and decreased physical functioning and mental health (11, 12). It has become evident that parenting a children with CHD affects the health-related quality of life (HRQoL) of the parents, which is defined as ‘a complex and multidimensional construct that captures individuals’ quality of life relative to their health or disease status, including symptoms, physical functioning, role functioning, and overall quality of life (13).

Previous researches demonstrated that CHD exerted a significant care burden on patients’ parents, leading to a lower HRQoL (14, 15). Khoshhal et al. (12) and Arafa et al. (16) reported the significantly poorer HRQoL in parents of children with CHD, who had lower scores in HRQoL, compared with parents of minor illnesses or healthy children. However, according to a study which aimed to evaluate parental quality of life among parents of infants with complex single ventricles, the overall parental quality of life scores were not significantly inferior to the established norms for a midwestern community sample of parents (17). Hua and Chen (18) concluded that the physical, psychological, and social domains of HRQoL were similar between parents of children with CHD and the general population. Obviously, these findings suggested that parents who raised a child with CHD showed conflicting results on HRQoL.

Over the past few decades, several studies have explored the factors that affect the HRQoL among parents of children with CHD, including symptoms of posttraumatic stress (15), perceived support, heart defect severity, age of the child, availability of economic resources (19), and psychoeducation (20). It has also been found that the HRQoL of mothers were lower than fathers, and mothers appeared to be particularly at risk for adverse HRQoL outcomes, implying that parent sex was another factor (15, 21, 22). In contrast, some studies have shown that gender has no effect on parents’ HRQoL. Bevilacqua et al. (23) supported that there was no meaningful difference between the state of mental health of women and men in parents of children with CHD. Mussatto et al. (24) published a study concerning the HRQoL of parents of children with CHD, which indicated that sex is not a factor influencing parents’ HRQoL.

Heart defect severity has been evidenced to decrease the HRQoL of parents of children with CHD in some researches (25, 26), while it has also been suggested that their HRQoL were not influenced by the complexity of CHD (27, 28). Furthermore, there may be some unknown factors that influence the HRQoL of parents of children with CHD. Since the HRQoL of parents strongly influences the health of the child, and a greater understanding of the key factors that influence HRQoL would facilitate the timely identification of parents who may be at risk (15), it is important to identify factors that influence the HRQoL of parents of children with CHD. In this systematic review and meta-analysis, we aimed to systematically examine the HRQoL of parents of children with CHD, and sought to determine the influencing factors.

## Materials and methods

### Design

This systematic review and meta-analysis was guided followed the Joanna Briggs Institute Manual for Evidence Synthesis and was registered with the International Prospective Register of Systematic Reviews (PROSPERO) with registration number CRD42024594518. We adhered the Preferred Reporting Items for Systematic Reviews and Meta-Analyses (PRISMA) reporting guideline.

### Search strategy and selection criteria

Systematic searches of Embase, PubMed, CINAHL, Web of Science, SCOPUS, China National Knowledge Infrastructure (CNKI), PsycINFO, Wanfang, Chinese Biomedical (Sinomed), Weipu (VIP) and SinoMed databases were conducted from inception to September 30, 2024. A snowballing method was also used to trace relevant references, aiming to ensure the comprehensiveness of the included literature. The search terms used a combination of Medical Subject Headings (MeSH) and free-text terms, and the search strategies used are presented in Supplementary File 1.

The studies were included if they fulfilled the following criteria: (1) the study population was composed of parents of children with CHD. (2) The outcomes reported included HRQoL or the influencing factors associated with the HRQoL of parents of children with CHD. (3) Full-text articles published in English or Chinese. (4) Original research articles in a cross-sectional, case–control, or cohort. The exclusion criteria included the following: (1) the study measured only 1 aspect of HRQoL (e.g., only mental health or social function). (2) A review, conference abstract, or case report. In addition, in cases where duplicate or repeated data appeared in multiple reports, the data with the most comprehensive information were included. If potentially eligible studies lacked outcome data, first, the corresponding authors were contacted to obtain the necessary information that was not included in the original articles.

### Study selection and data extraction

The initial database search results were imported into the EndNote X9 reference management software to remove duplicates and subsequently imported into the Joanna Briggs Institute Summary platform for literature screening and evaluation. Two reviewers (YW and JY) independently reviewed the titles, abstracts, and/or full manuscripts to determine study eligibility. In instances where discrepancies arose, two reviewers resolved them through discussion. If needed, discussions or consultations with the primary reviewer (FM) were employed to reach a consensus. Two independent reviewers (YW and JY) extracted the data using standardized data extraction forms, including the general characteristics (first author name, year of publication, region, and study design), clinical features (age, sex composition, sample size, scale, and types of congenital disease in children), and outcomes (measurement results and influencing factors).

### Risk of bias appraisal and quality assessment

The methodological quality of the cross-sectional studies included in the review was evaluated by two reviewers (W. Y and Y. J. R) using the Analytical Cross-Sectional Study Checklist provided by the Joanna Briggs Institute; studies that have 50% or more ‘Yes’ across the quality assessment parameters are considered low risk (29). The quality of cohort and case–control studies was evaluated via the Newcastle-Ottawa Scale (NOS), a widely recognized tool for assessing the quality of non-randomized studies (30). This scale assigns points on the basis of the appropriateness of participant selection (0–4 points), comparability (0–2 points), and exposure or outcome (0–3 points). A maximum of 9 points is assigned to each study, with a final score of 7 points or greater indicating high quality (31). The methodological quality of the studies was evaluated using a risk of bias grading system that categorized reviews as having low, high, or unclear risk. Any disagreements between the two reviewers were resolved through discussion. In instances where a consensus could not be reached, the primary reviewer (M. F.) was available for immediate consultation.

### Data synthesis and analysis

The extracted or calculated means, *SDs*, sample sizes, and 95% confidence intervals (*CIs*) were used to calculate pooled estimates of the HRQoL scores (32).

When evaluating continuous variables with non-harmonized assessment tools, the standardized mean difference (*SMD*) with 95% CI was employed to calculate the merged effects, and pooled effects were subsequently determined. The heterogeneity of the study was determined jointly via Cochrane’s *Q* test and the *I^2^* index. A fixed-effects model was employed for meta-analysis in the presence of *p* ≥ 0.05 and *I^2^* < 50%, whereas a random-effects model was used for effect size pooling in the event of *p* < 0.05 and *I^2^* ≥ 50% (33). Publication bias was assessed using the Egger’s test, and all the statistical analyses were performed using Stata 16.0. For all results, a two-sided *p*-value of 0.05 or less were considered significant. Subgroup analysis was performed based on the scales of HRQoL. As studies reported different types of effect sizes, all effect sizes were transformed to Pearson’s correlations and used Pearson’s correlation coefficient and 95% *CI* were used as the effect size of each influencing factor.

## Results

### Study selection and quality appraisal

**Figure 1:**
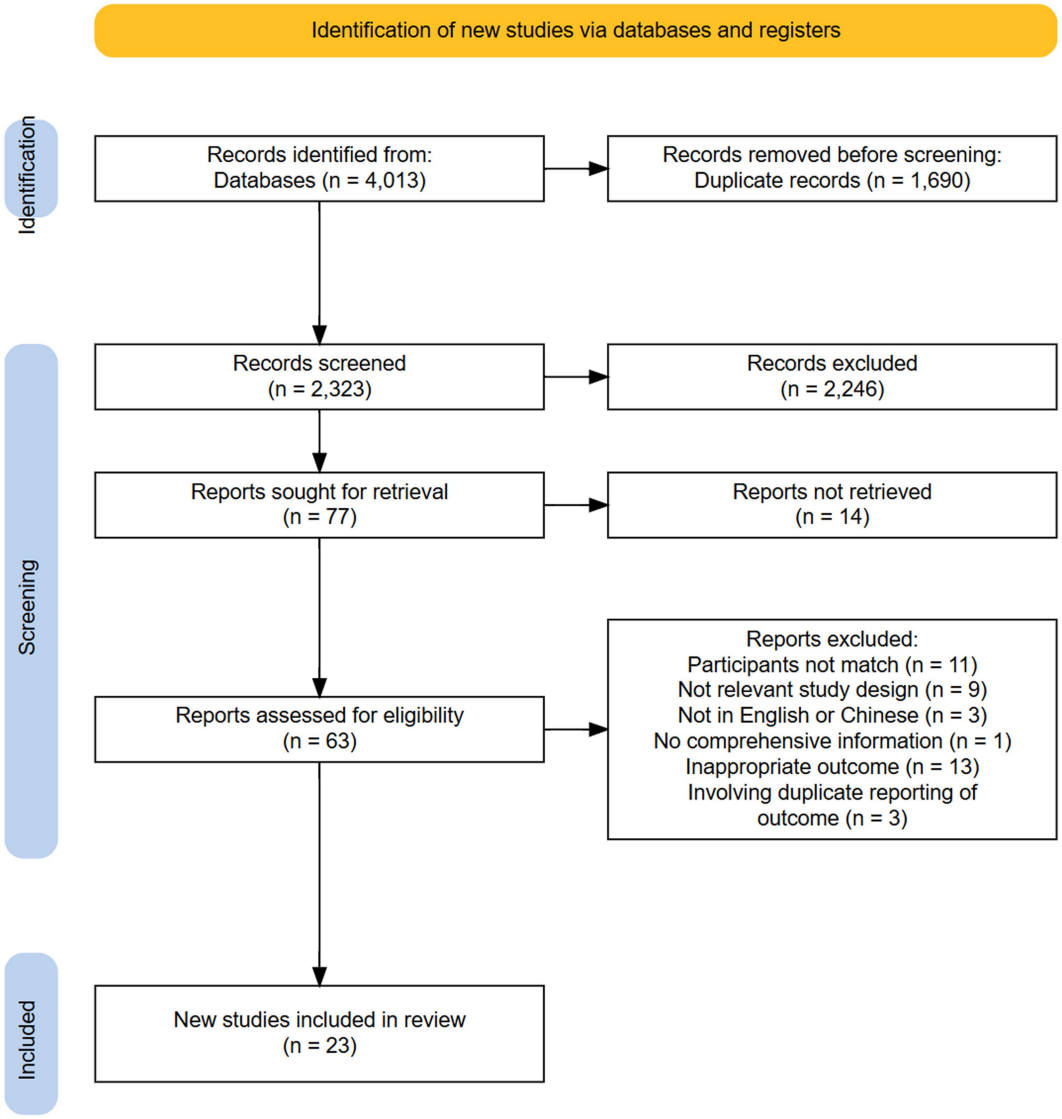
PRISMA flowchart of study selection.

The initial screening retrieved 4,013 articles, of which 1,690 of were duplicates; then we identified 2,323 abstracts. At the end of the title and abstract review, 77 reports were taken for full-text review by excluding the obviously irrelevant reports and reports that clearly did not comply with the prespecified eligibility criteria. After full-text review, a total of 23 observational studies were included in the systematic review, with cross sectional (*n* = 22), and cohort studies (*n* = 1) included in the systematic review, and no additional studies were added after the references were searched. All 22 cross-sectional studies scored ‘high’ quality using the JBI critical appraisal tool. With the use of the NOS, one study (34) received a 7-star rating, which denoted a satisfactory-quality article with a low risk of bias (Supplementary File 2). The screening process and the reasons for the exclusion of the articles are summarized in Figure 1.

### Study characteristics

The studies were conducted from 2003 to 2024. Geographically, nine studies were from Asia, five from South America, five from Europe, three from Oceania, and one from Africa. The included studies utilized 11 different scales to collect information on HRQoL, including the SF-12 (*n* = 1), SF-36 (*n* = 6), WHOQOL-BREF (*n* = 4), PedsQL FIM (*n* = 4), ULQIE (*n* = 2) and others (*n* = 6). These studies included a total of 36 influencing factors; if ≥2 studies mentioned the same influencing factor, meta-analysis will be conducted. Among all included studies, one study (35) had missing SD values, only one study (36) utilized the SF-12 scale and had not calculated total scores, one study (37) did not report HRQoL outcomes and influencing factors, and one study (38) reported only influencing factors that could not be included in the meta-analysis. Consequently, these discrepancies resulted in the inclusion of 19 studies in the subsequent meta-analysis, and four in the qualitative analysis (Table 1).

**Table 1:** Characteristics of included studies.

**Study ID**	**Publication year**	**Country**	**Design**	**Mean age**	**Sample(*n*)** **F-M-P**	**HRQoL Questionnaire**	**HRQoL Score Mean(*SD*)**	**Influencing factors**
Alkan (34)	2017	Turkey	cohort	36.8 (6.64)/M	0-80-0	SF-36^1^	PF:96 (12.66); SF:81.5 (15.02); RP:87.5 (27.54); RE:69.2 (28.46); MH:72.8 (15.04); VT:63.75 (16.26); BP:55.2 (6.57); GH:58.45 (11.29)	NA
Bevilacqua (23)	2013	Italy	Cross- sectional	33.3 (5.5)/M 36.4 (6.7)/F	38-36-0	SF-36^1^	State of mental health: mothers, 33.3 (13.8), fathers, 37.2 (12.2); State of physical health: mothers, 49.2 (9.5), fathers, 54.4 (6.9)	NA
Landolt (15)	2011	Switzerland	Cross- sectional	34.6 (5.4)/M 37.6 (6.5)/F	97-135-0	SF-36^1^	PF:97.84 (4.08); SF:73 (26.91); RP:80.12 (30.78); RE:73.09 (36.28); MH:69.05 (18.67); VT:49.69 (22.39); BP:85.24 (23.48); GH:76.84 (17.95)	1, 2, 3, 4, 5, 6
Liu (39)	2021	China	Cross- sectional	NA	0-0-115	SF-36^1^	PF:71 (22.5); SF:55 (28.2); RP:45 (31.3); RE:38 (15.2); MH:41 (21.1); VT:76 (32.8); BP:67 (17.5); GH:59 (26.6)	7, 8
Sileshi (40)	2017	Ethiopia	Cross- sectional	NA	0-135-0	SF-36^1^	PF:83.9 (22.8); SF:65 (29.5); RP:74.3 (53.7); RE:55.5 (42.8); MH:52.7 (19.1); VT:60.6 (25.7); BP:63.5 (26.5); GH:66 (26.2)	NA
Liao (41)	2018	China	Cross- sectional	NA	0-0-236	SF-36^1^	PF:85.59 (13.14); SF:70.39 (23.19); RP:63.14 (40.37); RE:55.93 (41); MH:58.72 (18.86); VT:62.27 (20.10); BP:77.11 (19.43); GH:63.89 (20.7); Total:67.13 (17.70)	9, 10, 11, 12
Bektas (38)	2020	Turkey	Cross- sectional	34.83 (7.87)/P	10-114-0	WHOQOL-BREF^2^	NA	13, 14
Khoshhal (12)	2019	Saudi Arabia	Cross- sectional	36.8 (9.4)/P	0-0-120	WHOQOL-BREF^2^	Physical:11.42 (3.02) Psychological:9.84 (2.42) Social:10.78 (3.27) Environmental:11.24 (2.62) Total:52.84 (11)	NA
Lin (42)	2024	China	Cross- sectional	28.87 (5.8)/P	0-0-81	WHOQOL-BREF^2^	Physical:9.98 (1.60) Psychological:13.58 (1.90) Social:11.28 (1.70) Environmental:10.47 (2.46) Total: 45.28 (4.3)	15, 16, 17
Coban (5)	2022	Turkey	Cross- sectional	32.3/P	0-0-60	WHOQOL-BREF^2^	Physical: 13.30 (2.60) Psychological:14.23 (2.98) Social:14.22 (3.70) Environmental:13.59 (2.53)	NA
Eagleson (25)	2012	Australia	Cross- sectional	NA	0-0-60	PedsQL FIM^3^	Total:78.43 (19.92)	NA
Denniss (43)	2019	Australia	Cross- sectional	34.0 (5.8)/M	0-84-0	PedsQL FIM^3^	Total:71.88 (19.53)	18, 19, 20, 21
Kaugars (44)	2018	USA	Cross- sectional	NA	0-0-54	PedsQL FIM^3^	Total:71.96 (22.54)	22
Lee (28)	2020	Canada	Cross- sectional	NA	74-66-140	PedsQL FIM^3^	Total:91.11 (12.38)	NA
Goldbeck (45)	2006	Germany	Cross- sectional	NA	0-0-132	ULQIE^4^	Total:75.2 (14.7)	NA
Golfenshtein (46)	2023	USA	Cross- sectional	30.32 (5.66)/P	4-86-90	ULQIE^4^	Total:71.36 (9.54)	23
Casey (27)	2024	Ireland	Cross- sectional	36.77 (4.56)/P	0-0-51	Quality of Life for Parents and their Children with CHD	Total:70.14 (23.23)	12, 24, 25
Mussatto (24)	2023	USA	Cross- sectional	35.56 (5.87)/M 38.23 (7.42)/F	0-0-192	PQoL^5^	Total:7.4 (1.3)	17, 24
Utens (35)	2016	Netherlands	Cross- sectional	NA	85-76-149	Linear Analogue Scale	Total:89.45	NA
Liang (37)	2022	China	Cross- sectional	34.83 (7.87)	0-0-188	Quality of life	NA	NA
Lawoko (14)	2003	Sweden	Cross- sectional	39 (7)	420-664-1085	The Quality of Life Scale	Physical:14.5 (0.10) Psychological:18.2 (0.14) Total:68 (0.43)	16, 17, 25, 26, 27, 28, 29, 30, 31, 32, 33, 34, 35, 36
Azhar (26)	2016	Saudi Arabia	Cross- sectional	NA	19-157-176	Self-made quality of life questionnaire.	Biological impact score: 21.07 (24.73) Psychological impact score: 19.93 (17.20) Social impact score: 13.90 (15.96) Global impact score:18.21 (12.33)	NA
Delaney (36)	2023	USA	Cross- sectional	27 (4)	5-18-23	SF-12^6^	MH:36.94 (13.26); PH:51.09 (7.40)	NA

1 Short Form-36 Health Survey; ^2^World Health Organization Quality of Life-Brief Version; ^3^Pediatric Quality of Life Inventory Family Impact Model; ^4^Ulm Quality of Life Inventory; ^5^Perceived Quality of Life; ^6^Short Form-12 Health Survey.

Influencing factors: (1)mothers: lower socioeconomic status; (2)mothers: nonswiss nationality; (3)mothers: full or partial PTSD at discharge; (4)mothers: current impact on family; (5) fathers: full or partial PTSD at discharge; (6) fathers: current impact on family; (7) positive religious coping; (8) negative religious coping; (9) anxiety; (10) depression; (11) positive coping; (12) negative coping; (13)care burden; (14) symptom frequency of the child; (15) religious belief; (16) age (parents); (17) Gender; (18) mothers: presence of comorbidity; (19) mothers: family functioning; (20) mothers: maternal psychological stress; (21) mothers: difficult child temperament; (22) difficulty to care for a child with CHD; (23) infant temperament; (24) length of hospital stay under cardiology; (25) CHD complexity; (26) Other disease (not CHD); (27) child gender; (28) number of CHD diagnosis; (29) number of operations; (30) child age; (31) care-giving time; (32) education: secondary, university; (33) current employment: sick-leave, pension; (34) concerns about finances; (35) difficulties with living expenses; (36) financial burden of CHD.

### Results of the meta-analysis of the HRQoL scores

**Figure 2:**
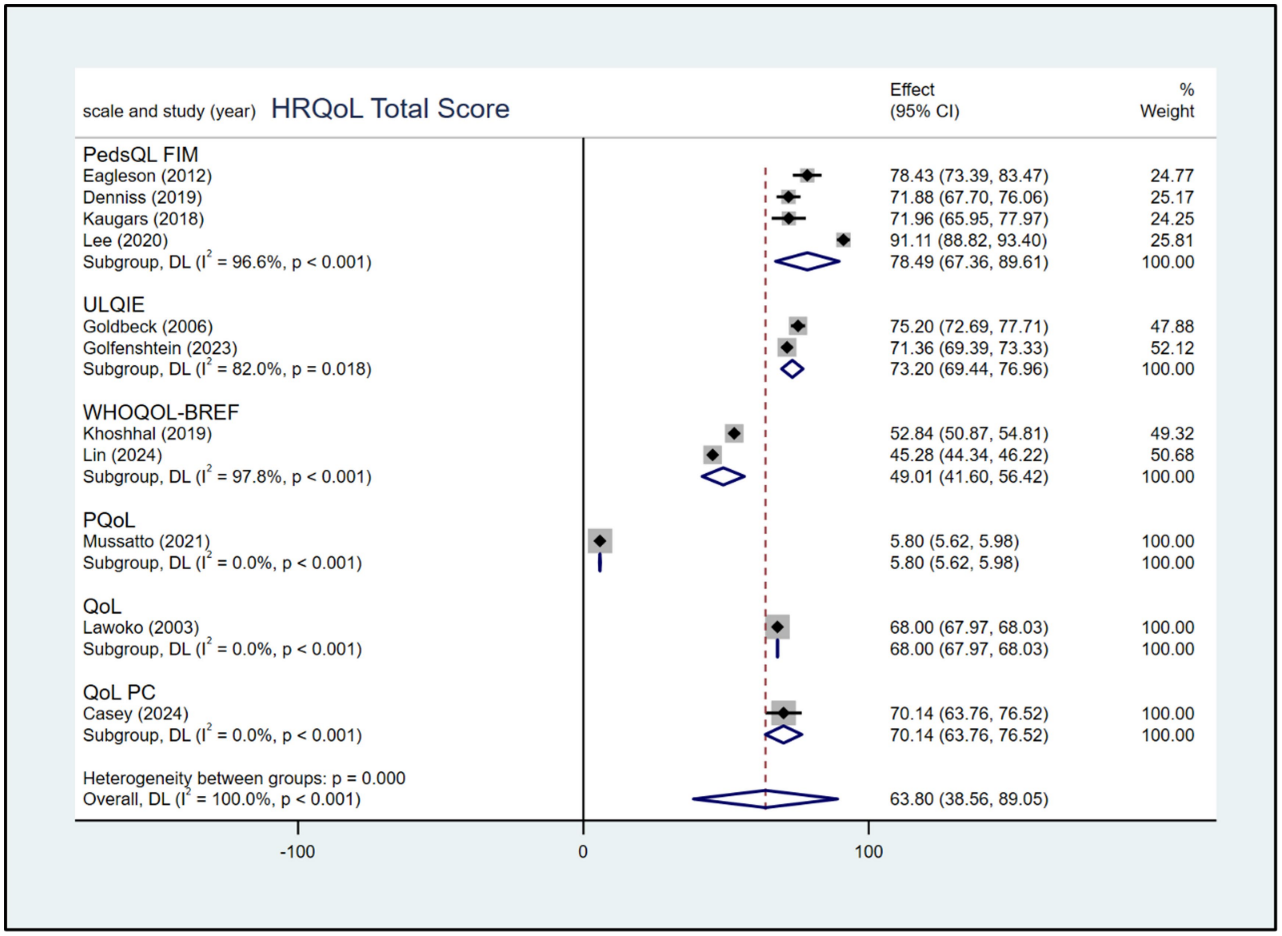
Forest plot, based on subgroup analysis based on the scale, of the pooled scores of HRQoL among parents of children with CHD.

According to the different scales used, the meta-analysis results revealed the different pooled scores of HRQoL among parents of children with CHD. The meta-analysis showed a pooled of PedsQL score was 78.49 (95% CI: 67.36, 89.61), ULQIE score was 73.20 (95% CI: 69.44, 76.96), WHOQOL-BREF score was 49.01 (95% CI: 41.60, 56.42). The forest plot is presented in Figure 2.

**Figure 3:**
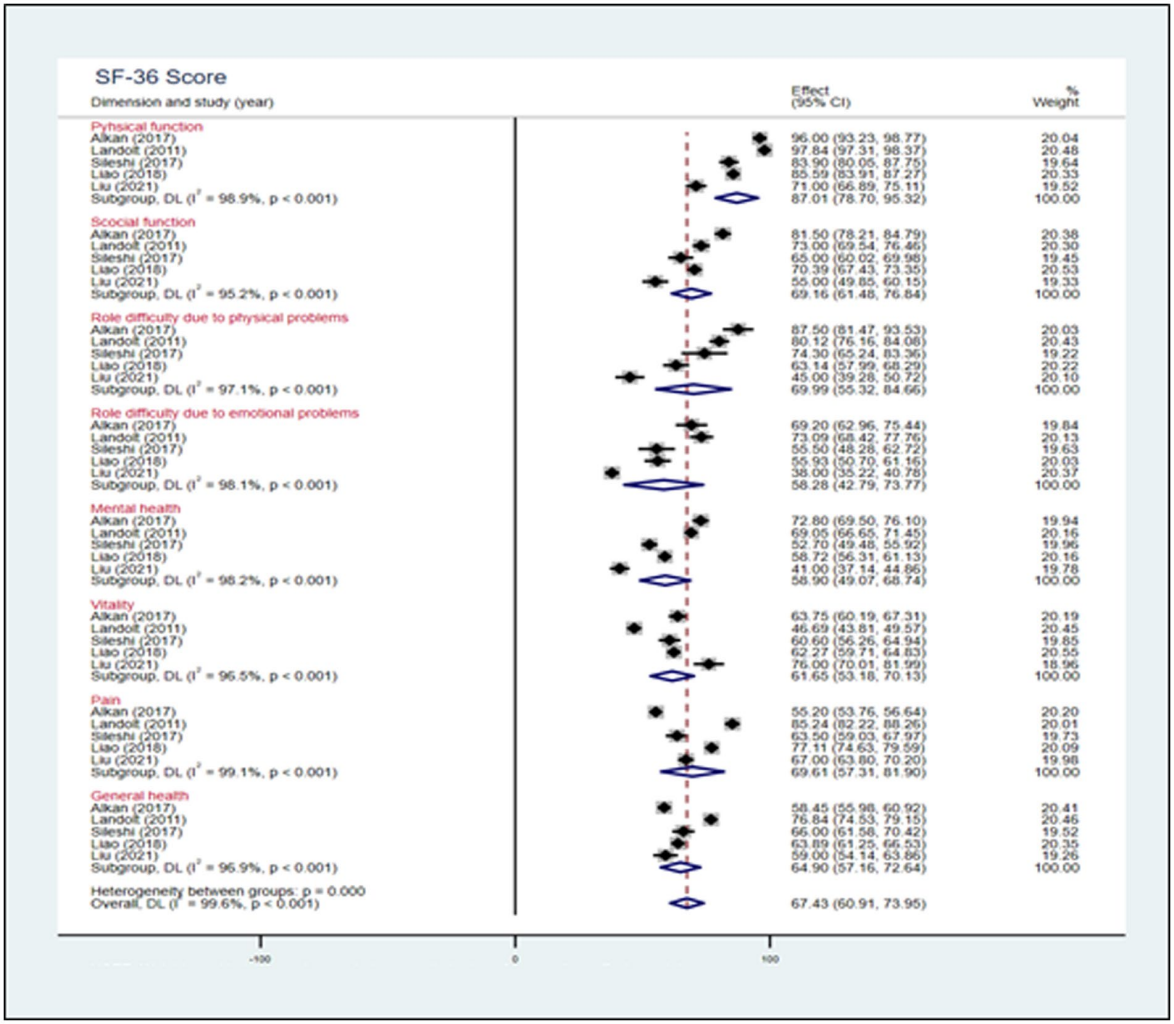
Forest plot, analysis of the pooled scores of HRQoL among parents of children with CHD based on 8 domains of the SF-36 scale.

Figure 3 shows that five studies were included in the calculation of pooled scores for eight domains of the SF-36 scale among parents of children with CHD. The meta-analysis showed pooled scores of 64.90(95% CI: 57.16, 72.64) in the general health dimension, 58.90(95% CI: 49.07, 68.74) in the mental health dimension, 69.61(95% CI: 57.31, 81.90) in the pain dimension, 87.01(95% CI: 78.70, 95.32) in the physical function dimension, 58.28(95% CI: 42.79, 73.77) in the role difficulty due to emotional problems dimension, 69.99 (95% CI: 55.32, 84.66) in the role difficulty due to physical problems dimension, 69.16 (95% CI: 61.48, 76.84) in social function dimension, and 61.65 (95% CI: 53.18, 70.13) in the vitality dimension.

**Figure 4:**
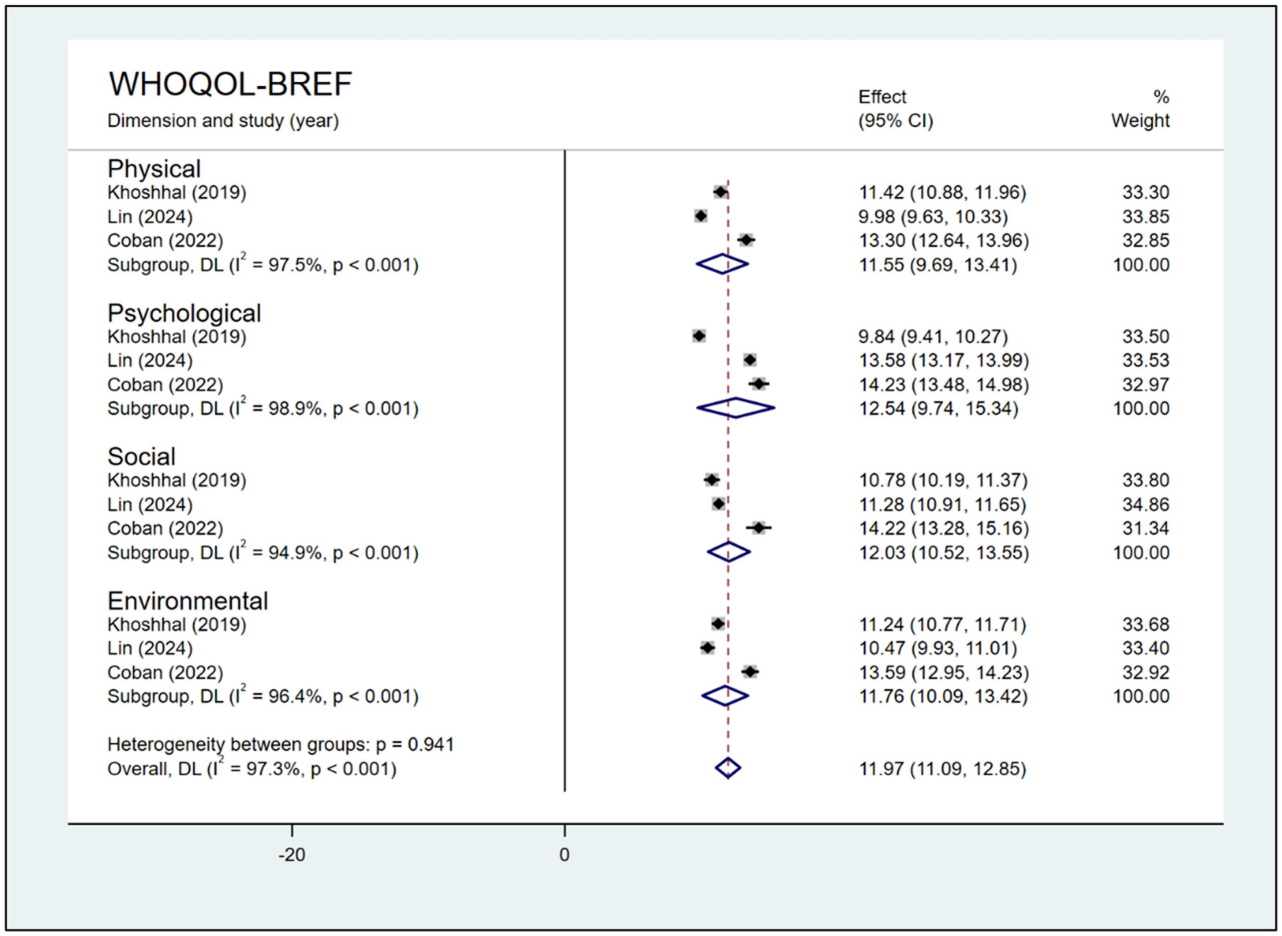
Forest plot, analysis of pooled scores of HRQoL among parents of children with CHD based on the 4 domains of the WHOQOL-BREF.

Three studies used the WHOQOL-BREF to evaluate HRQoL among parents of children with CHD. The meta-analysis showed scores of 11.55 (95% CI: 9.69, 13.41) in the physical dimension, 12.54 (95% CI: 9.74, 15.34) in the psychological dimension, 12.03 (95% CI: 10.52, 13.55) in the social dimension, and 11.76 (95% CI: 10.09, 13.42) in the environmental dimension (Figure 4).

**Figure 5:**
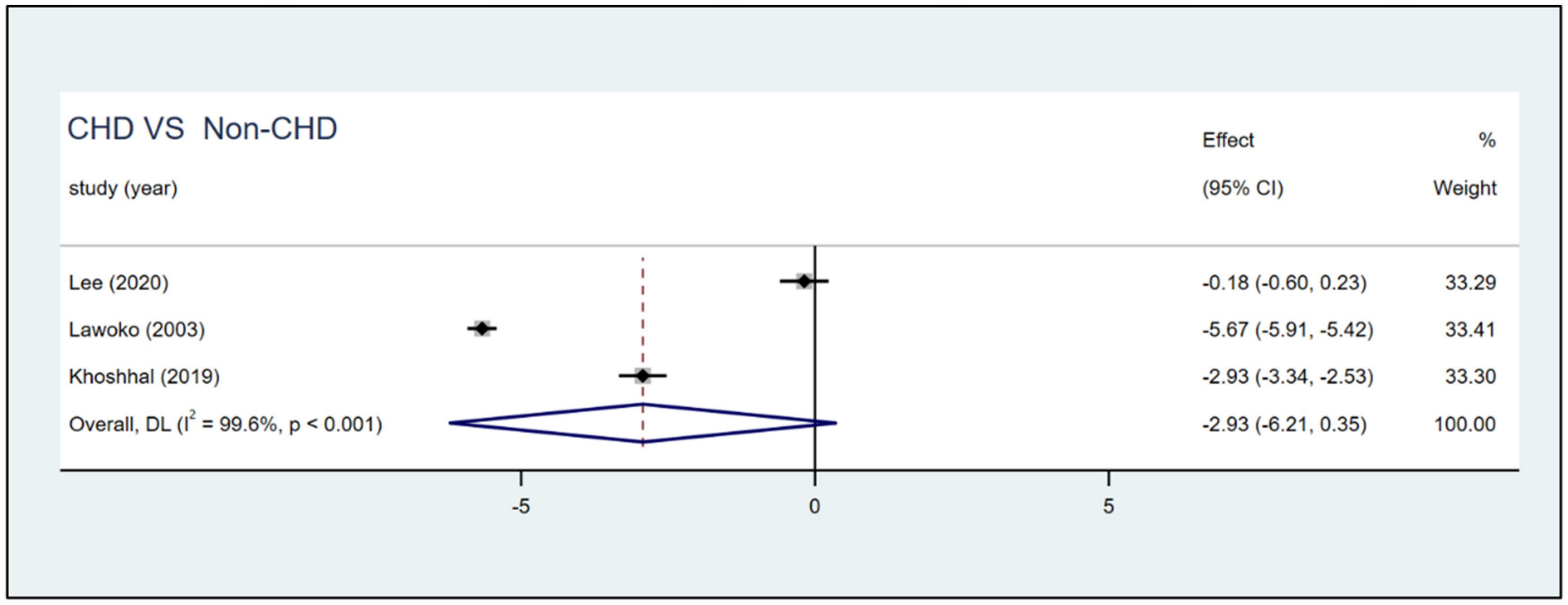
Forest plot, and meta-analysis of the HRQoL score among parents of children with CHD and nonprivate children with CHD across different studies.

**Figure 6:**
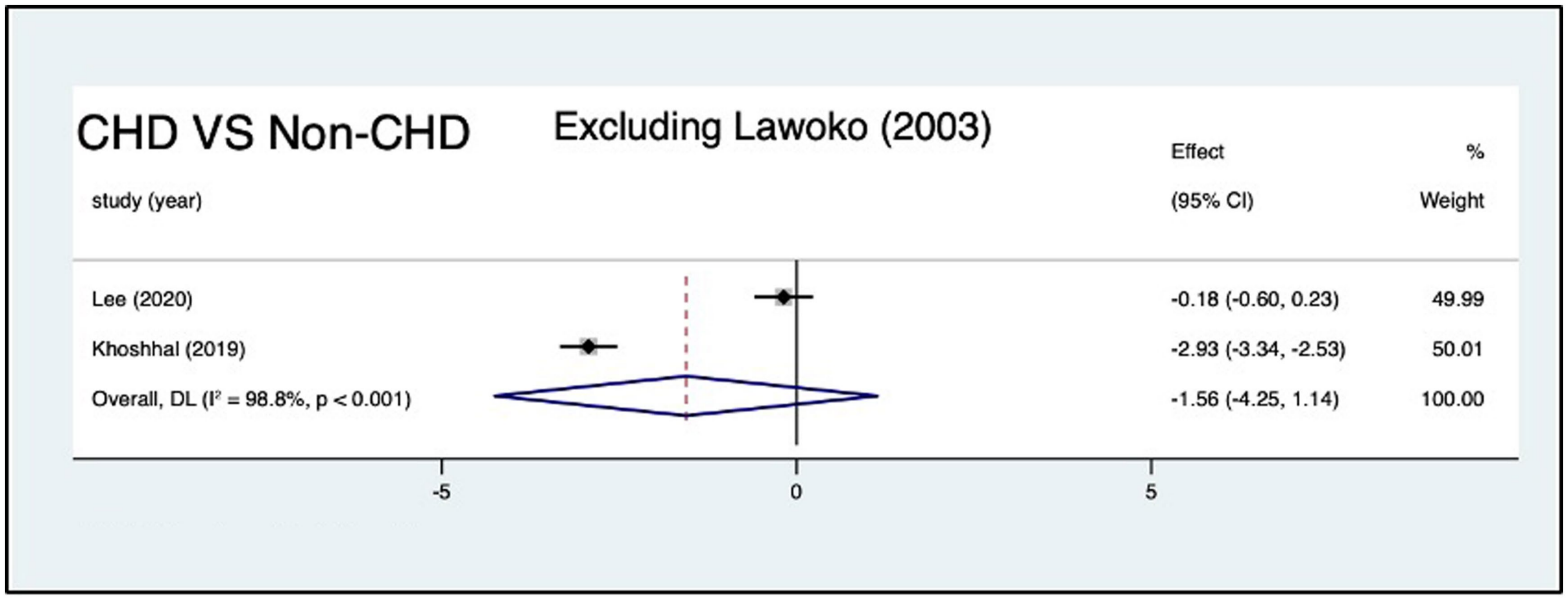
Forest plot, exlcude outlier.

Three studies compared HRQoL between parents of children with CHD (*n* = 1,317) and those without CHD (*n* = 397). Compared with parents of children without CHD, parents of children with CHD had no significant different in total score [*SMD*: −2.93 (95% CI: −6.21, 0.35); *p* = 0.08; *I*^2^: 99.6%; *p* < 0.001; random effects model] (Figure 5). As the extreme effect size from Lawoko and Soares (14) SMD = −5.67 (95% CI: −5.91, −5.42); deviated by >2 SD from other studies (Figure 5). We excluded the study to do sensitivity analyses, and the result revealed that the pooled SMD changed from −2.93 (95% CI: −6.21, 0.35) to −1.56 (95% CI: −4.25, 1.14), *I^2^* decreased from 99 to 98.8% (Figure 6).

Two studies used the SF-36 scale to compare HRQoL between mothers of children with CHD (*n* = 215) and those without this condition (*n* = 175); the meta-analysis results are shown in Table 2.

**Table 2:** Meta-analysis of SF-36 Scale Score among mothers of children with CHD versus mothers of children without CHD (*n* = 3).

**SF-36 Scales Dimension**	**parents of children with CHD (*n*)**	**parents of children without CHD** **(*n*)**	***SMD* (95% *CI*)**	***p***	***I*** ^***2***^ **(95% *CI*); *P* value**	**Effects model**
Physical function	215	175	-0.25(-0.57,0.07)	0.123	51.8%; *P* = 0.150	random
Social function	215	175	-0.53(-0.74,-0.33)	<0.001	30.5%; *P* = 0.230	fixed
Role difficulty due to physical problems	215	175	-0.31(-0.52,-0.11)	0.003	0.0%; *P* = 0.505	fixed
Role difficulty due to emotional problems	215	175	-0.79(-1.00,-0.58)	<0.001	9.2%;*P* = 0.505	fixed
Mental health	215	175	-0.89(-2.37,0.59)	0.239	97.5%; *P* < 0.001	random
Vitality	215	175	-0.55(-1.27,0.16)	0.128	89.8%; *P* = 0.002	random
Pain	215	175	-0.43(-0.90,0.04)	0.074	77%; *P* = 0.037	random
General health	215	175	-0.58(-0.79,-0.37)	<0.001	23.8%; *P* = 0.252	fixed

### Influencing factors of HRQoL in parents of children with CHD

**Figure 7:**
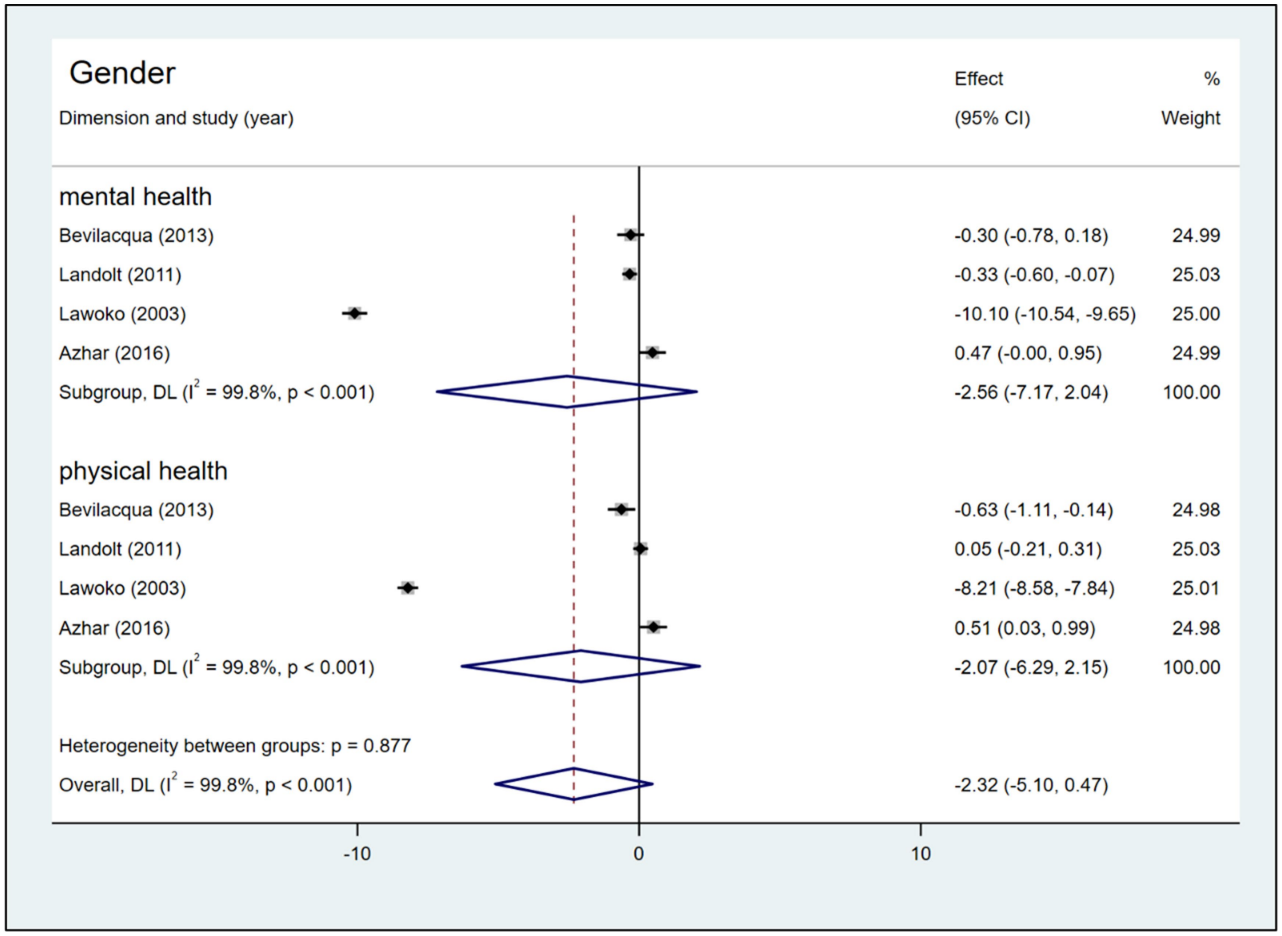
Forest plot, and meta-analysis of HRQoL score between mothers and fathers of children with CHD.

**Figure 8:**
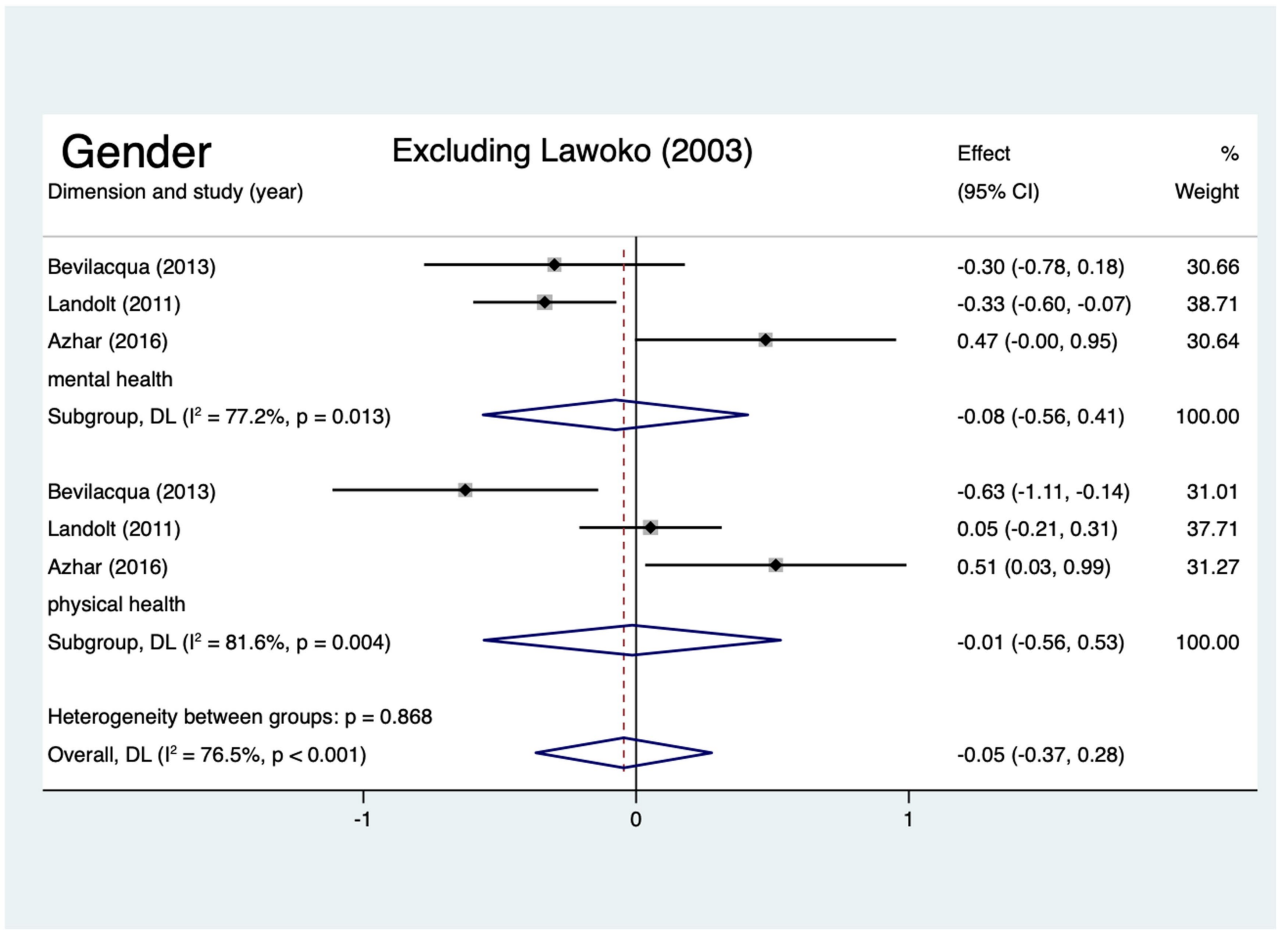
Forest plot, exclude outlier.

Four articles, which were included in the meta-analysis, assessed the mental health and physical health of HRQoL of mothers and fathers of children with CHD. The scores for the two domains did not differ significantly among mothers and fathers (Figure 7). Egger’s test revealed no publication bias was observed for the physical health or mental health scores (*p* > 0.05). Lawoko and Soares (14) SMD = −10.10 (95% CI: −10.54, −9.65) in the dimension of mental health and SMD = −8.21 (95% CI: −8.58, −7.84) in the dimension of physical health deviated by >2 SD from other studies (Figure 7). Excluding the study, the results showed that the conclusion remains unchanged, but in dimension of mental health the pooled SMD changed from −2.56 (95% CI: −7.17, 2.04) to −0.08 (95% CI: −0.56, 0.41), *I^2^* decreased from 99.8 to 77.2%, and in dimension of physical health the pooled SMD changed from −2.07 (95% CI: −6.29, 2.15) to −0.01 (95% CI: −0.56, 0.53), *I^2^* decreased from 99.8 to 81.6% (Figure 8).

Among the 36 influencing factors, five contributed to the pooled effect size. The remaining influencing factors could not be pooled because only one study could obtain data. Two pieces of literature analyzed the influence of negative coping on the HRQoL of parents of children with CHD, and the meta-analysis results showed a statistically significant relationship between negative coping and the HRQoL of parents of children with CHD However, the remaining all influencing factors were not statistically significant related to the HRQoL of parents of children with CHD (Table 3).

**Table 3:** Influencing factors of HRQoL of parents of children with CHD: a meta-analysis.

Influencing factors	Number of studies	Heterogeneity test		Effects model	Pooled effect size		
		*I^2^*	*P*		Fishers’ *Z* (95% CI)	*P*	*r*
Negative coping	2	87.6%	0.005	Random	−0.70 (−1.16, −0.25)	0.003	−0.60 (−0.82, −0.24)
Gender	3	90.7%	<0.001	Random	0.25 (−0.003, 0.50)	0.053	0.24 (−0.003, 0.46)
Total hospital length of stay	2	77.9%	0.033	Random	−0.16 (−0.49, 0.17)	0.350	−0.16 (−0.45, 0.17)
Parental age	2	63.0%	0.100	Random	0.14 (−0.04, 0.31)	0.137	0.14 (−0.04, 0.30)
CHD complexity	2	71.5%	0.061	Random	0.008 (−0.18, 0.34)	0.538	0.08 (−0.18, 0.33)

### Sensitivity analysis and publication bias

Sensitivity analysis was performed using fixed- and random-effects models and using the one-by-one exclusion method. Using the Egger’s test to sensing the publication bias. The total analysis results are shown in Supplementary File 3.

## Discussion

This systematic review and meta-analysis summarized data about the HRQoL and influencing factors of parents of children with CHD. Our study demonstrated that mothers of children with CHD have been shown to exhibit lower social function and general health, as well as greater role difficulty due to emotional and physical problems when compared to the general population. This can be attributed to the fact that mothers exhausted nearly all of their time in providing care for the children with CHD (39), rarely prioritized their own interests and social activities, even have to abandon their jobs (40, 41). Furthermore, the high costs of medical treatment expenditures (42), suffering from social isolation, and loss of social roles make mothers feel hugely stressed and deteriorate their health (43, 44). Therefore, future studies are needed to accurately identify the HRQoL of mothers and to plan and devise a strategy to support these mothers (45, 46).

However, it is noteworthy that SF-36 scores did not significantly differ in mental health, physical function, vitality, and pain domains between mothers of children with CHD and the general population. This finding is inconsistent with those of previous studies, which have suggested that mothers of children with CHD experience a variety of mental health problems (e.g., psychological distress, anxiety, somatization, and posttraumatic stress symptoms) (21, 47, 48), vitality, and physical functioning problems (e.g., fatigue and loss of energy) (16). A possible explanation for this observation is that mothers of children with CHD undergo a progressive process of extrication from ‘mom guilt’ and accepting existential guilt, which can be seen as a drive toward self-repair, manifesting in the form of receptivity and openness to a future life (49, 50). Consequently, mothers of children with CHD would no longer attempt to attribute their children’s heart defects to themselves, and they would no longer use this as a means of measuring their own happiness. They protect the child with courage, face challenges with equanimity, overcome adversity through positivity and gratitude (51), and access self-fulfillment from caring for CHD children (52).

Interestingly, previous systematic reviews (19, 53, 54) have revealed that there existed a divergence in the HRQoL of parents whose children were afflicted with CHD and those whose children were afflicted with minor illnesses or were healthy children. However, our results have revealed a non-significant difference in total HRQoL scores. The explanations for this phenomenon are that parents of children with CHD might develop family resilience across time, and the family-centered care models are evolving. In the process of the disease, families might be able to adapt to stress and recover from adversity, fulfill patients’ parental roles, derive joy from parenting, and experience a positive change, which might improve their HRQoL (51, 55, 56). Additionally, family-centered care that addresses the expectations and needs of parents is associated with improved parents’ health and may contribute to the health of the entire family system (57–59).

The single most striking to emerge from our results was that, regardless of the mental or physical health dimension and even in the total HRQoL scores, there were no statistically significant differences between fathers and mothers who had a CHD children. This finding is inconsistent with the findings of past studies. Kolaitis et al. (47) and Cole et al. (7) reported that mothers had more problems with physical and emotional functioning limitations and experienced lower HRQoL compared with fathers of children with CHD. Here, we turn our interpretation toward a sociological perspective. Fathers of children with CHD need to fulfill the fatherhood (60), and in the culture of fatherhood, there has been a notable shift, which is the ‘new fathers’ of today, which has entailed higher expectations for father involvement in the care of young children, including more nurturing, closer emotional relationships with their children, and sharing the joys and work of caregiving with mothers (61).

These ‘new fathers’ are presented as being just as capable as mothers with respect to child rearing, which has led researchers to suggest that we are “moving toward a social ideal of father as co-parent” (61, 62). Fathers of children with CHD can now recognize their partners’ stress, communicate effectively with them, and rely more closely on and support each other (63, 64), which may attenuate mothers’ daily problems (65), facilitate mothers’ relationship quality (66) and help improve mothers’ HRQoL. Furthermore, fathers assume twofold responsibilities, including being worried about their wives as well as their children, and they have to balance employment with care for their family (44, 67, 68), which might decrease their HRQoL. However, only four studies provided date from mothers and fathers data and employed different scales (e.g., the SF-36 and the Self-made quality of life questionnaire) to assess HRQoL, and inconsistent results in sensitivity analyses; thus, the results should be interpreted with caution.

It is worth discussing the outlier of this review. When we excluded the study by Lawoko and Soares (14), we found that the pooled effect size, the standard errors and confidence interval of the pooled effect size, and *I^2^* had changed. Probably, these discrepancies emerged because of selection bias, a small control group. The study used members of the Swedish Heart Children’s Foundation (SHCF) as participants, which might limit representativeness, as not all parents of children with CHD are SHCF members (4). In addition, imbalanced group sizes (healthy children’s parents = 289 vs. CHD parents = 1,085) may reduce generalizability and statistical power, contributing to heterogeneity in meta-analyses. Sensitivity analysis showed that removing this study did not alter our conclusions; meanwhile, it reduced heterogeneity and narrowed the confidence intervals, further strengthening the robustness of our findings.

This meta-analysis revealed that negative coping as a potential influencing factor of HRQoL among parents of children with CHD. Parental coping refers to parents’ ability to adjust to their baby’s CHD, fulfill their parental responsibilities and managing parental stress that emerges in medical and child-related scenarios (27, 69). Previous research has generally shown that negative coping, including hypervigilance, avoidance, denial, or distraction, is associated with poorer HRQoL outcomes (69, 70). For instance, one study (71) noted that negative religious coping was related to the HRQoL of the parents of infants with CHD, and it increased their psychological distress. Therefore, we recommend that interventions tailored to the needs of parents of children with CHD are needed (72), such as the congenital heart disease intervention program (CHIP)-family intervention or nursing education, which uses various media to improve mental health and coping mechanisms for parents of children with CHD (73). Furthermore, there is insufficient evidence to establish an association between four other factors, including gender, total hospital length of stay, parental age, and CHD complexity. These factors might have been influenced by the measurement instrument, sample size, and the limited number of included studies. Further research is warranted to explore these factors comprehensively.

The substantial statistical heterogeneity observed in this meta-analysis (e.g., *I^2^* = 99.6%) warrants careful interpretation of the pooled estimates. Study indicates that heterogeneity among studies affects the standard errors and confidence interval of the pooled effect size (74). However, the presence of heterogeneity does not render the meta-analysis results unimportant or invalid, which is a critical but unavoidable aspect of meta-analyses (75). Therefore, in clinical practice, healthcare professionals should interpret HRQoL scores with caution, given the substantial variability among assessment tools. Furthermore, developing standardized HRQoL instruments with robust psychometric properties for cross-population use could help reduce measurement heterogeneity and enhance comparability across studies.

### Strengths and limitations

This systematic review has several strengths, particularly regarding methods. First, this is the very first time that a meta-analysis has been performed to measure the HRQoL of parents of children with CHD as well as the influencing factors. Second, new insights into the HRQoL for parents of children with CHD could result from this research.

Several limitations should be noted. Firstly, our findings were affected, partly due to the heterogeneity in measurement tools used across studies, which lack of standardization of outcome variables and make interpretation of results across studies difficult. Secondly, the number of studies included in the meta-analysis was relatively small, and some of these studies also had small sample sizes and a disproportionate female-to-male ratios; thus, they may not represent the wider population. Thirdly, we regret not being able to perform more influencing factor analyses, because of the number of published influencing factors was so limited that it was simply not possible to perform a meta-analysis. Given these limitations, the key point of this study is that there is a need to focus on the HRQoL of parents of children with CHD, as well as on the possible risk factors involved and further in-depth studies are necessary to validate and expand upon the outcomes of this study in the future.

## Conclusion

Our review and meta-analysis demonstrated that mothers of children with CHD had lower social function and general health, and greater role difficulty due to emotional and physical problems than the general population, and our review revealed that there are a diverse number of instruments used to measure HRQoL in parents of children with CHD; a standardized approach to measure HRQoL is needed. It is noteworthy that SF-36 scores did not significantly differ in mental health, physical function, vitality, and pain domains between mothers of children with CHD and the general population Our results revealed that, regardless of the mental or physical health dimensions scores and even the total HRQoL scores, there were no statistically significant differences between fathers and mothers who had a CHD child. Additionally, although the results illustrated the association of negative coping with the HRQoL of parents of children with CHD, this result needs to be interpreted with caution.

## Data Availability

The original contributions presented in the study are included in the article/Supplementary material, further inquiries can be directed to the corresponding author.
