# Supplementary material for: Health-related quality of life and influencing factors in parents of children with congenital heart disease: a systematic review and meta-analysis
Source: Front Public Health. 2025 Oct 10;13:1622491. doi: 10.3389/fpubh.2025.1622491 (PMC12550949; doi:10.3389/fpubh.2025.1622491)
Supplement: Supplementary file 1 [file Supplementary_file_1.docx]

Search strategy (Till September 30, 2024)

**Pubmed**

610results

("congenital heart disease"[Title/Abstract] OR "congenital heart defect"[Title/Abstract] OR "congenital heart defects"[Title/Abstract] OR "congenital cardiac"[Title/Abstract] OR "cardiac defect congenital"[Title/Abstract] OR "CHD"[Title/Abstract] OR "heart defects, congenital"[MeSH Terms]) AND ("Parents"[MeSH Terms] OR ("parent"[Title/Abstract] OR "Parents"[Title/Abstract] OR "mother"[Title/Abstract] OR "mothers"[Title/Abstract] OR "father"[Title/Abstract] OR "fathers"[Title/Abstract] OR "mom"[Title/Abstract] OR "dad"[Title/Abstract] OR "maternal"[Title/Abstract] OR "paternal"[Title/Abstract] OR "parental"[Title/Abstract] OR "family"[Title/Abstract])) AND ("Quality of Life"[Title/Abstract] OR "health related quality of life"[Title/Abstract] OR "QOL"[Title/Abstract] OR "HRQOL"[Title/Abstract] OR "well-being"[Title/Abstract] OR "well-being"[Title/Abstract] OR "life satisfaction"[Title/Abstract] OR "life enjoyment"[Title/Abstract] OR "Quality of Life"[MeSH Terms])

**Embase**

873results

'congenital heart disease'/exp OR 'congenital heart disease' OR 'congenital heart defect'/exp OR 'congenital heart defect' OR 'congenital cardiac' OR 'cardiac defect congenital' OR 'chd' AND 'quality of life'/exp OR 'quality of life' OR 'health related quality of life'/exp OR 'health related quality of life' OR 'qol' OR 'hrqol' OR 'well-being'/exp OR 'well-being' OR 'life satisfaction'/exp OR 'life satisfaction' OR 'life enjoyment' AND 'parents'/exp OR 'parent' OR 'mother'/exp OR 'father' OR 'mom' OR 'dad' OR 'parental' OR 'paternal' OR 'family'/exp '

**Scopus**

276results

( TITLE-ABS-KEY ( "congenital heart disease" ) OR TITLE-ABS-KEY ( "congenital heart defect" ) OR TITLE-ABS-KEY ( "congenital cardiac" ) OR TITLE-ABS-KEY ( "cardiac defect congenital" ) OR TITLE-ABS-KEY ( "CHD" ) ) AND ( TITLE-ABS-KEY ( "quality of life" ) OR TITLE-ABS-KEY ( "health related quality of life" ) OR TITLE-ABS-KEY ( "HRQOL" ) OR TITLE-ABS-KEY ( "well-being" ) OR TITLE-ABS-KEY ( "life satisfaction" ) ) AND ( TITLE-ABS-KEY ( "parents" ) OR TITLE-ABS-KEY ( "parent" ) OR TITLE-ABS-KEY ( "mother" ) TITLE-ABS-KEY ( "father" ) OR TITLE-ABS-KEY ( "maternal" ) OR TITLE-ABS-KEY ( "paternal" ) OR TITLE-ABS-KEY ( "parental" ) OR TITLE-ABS-KEY ( "family" ) )

**Web of Science**

1849 results

((((TS=(congenital heart disease)) OR TS=(congenital heart defect)) OR TS=(congenital cardiac)) OR TS=(cardiac defect congenital)) OR TS=(CHD) AND ((((((TS=(quality of life)) OR TS=(health related quality of life)) OR TS=(QOL)) OR TS=(HRQOL)) OR TS=(well-being)) OR TS=(life satisfaction)) OR TS=(life enjoyment) AND (((((((TS=(parent)) OR TS=(parents)) OR TS=(mother)) OR TS=(father)) OR TS=(parental)) OR TS=(maternal)) OR TS=(paternal)) OR TS=(family)

**CINAHL**

232 results

(congenital heart disease or cardiac defect or congenital heart defect or CHD) AND (quality of life or well being or well-being or health-related quality of life or life satisfaction or life enjoyment or HRQOL) AND (parent or parents or parental or mother or father or paternal or family)

**PsycINFO**

145 results

congenital heart disease OR congenital heart defect OR congenital cardiac OR cardiac defect congenital OR CHD CHD AND quality of life OR health related quality of life OR HRQOL OR well-being OR life satisfaction OR life enjoyment AND parent OR parents OR father OR mother OR maternal OR paternal OR parental OR family

**CNKI**

3 results

(主题:先天性心脏病)OR(关键词:先天性心脏畸形)OR(关键词:先天性心脏病缺陷)OR(关键词:先心病)AND(主题:健康相关生活质量)OR(关键词:生活质量)OR(关键词:健康生活质量) OR(关键词:生活质量与健康)AND(主题:父母)OR(关键词:父母)OR(关键词:爸爸)OR(关键词:妈妈)OR(关键词:父亲)OR(关键词:母亲)OR(关键词:双亲)

**Wanfang**

7 results

题名或关键词:(先天性心脏病 or 先天性心脏畸形 or 先天性心脏缺陷 or 先天性心脏血管疾病) and 题名或关键词:(健康相关生活质量 or 健康相关生命质量 or 健康相关生存质量 or 生活质量) and 题名或关键词:(父母 or 母亲 or 父亲 or 爸爸 or 妈妈 or 双亲)

**VIP Database**

0 results

(((((((题名或关键词=先天性心脏病 OR 题名或关键词=先心病) OR 题名或关键词=先天心脏病) OR 题名或关键词=先天性心血管病) OR 题名或关键词=先天性心脏) OR 题名或关键词=先天性心脏血管病) AND (((((题名或关键词=健康相关生活质量 OR 题名或关键词=健康相关生命质量) OR 题名或关键词=健康相关生存质量) OR 题名或关键词=健康相关生活品质) OR 题名或关键词=与健康相关的生存质量) OR 题名或关键词=健康生命质量)) AND (((((题名或关键词=父母 OR 题名或关键词=爸爸) OR 题名或关键词=妈妈) OR 题名或关键词=父亲) OR 题名或关键词=母亲) OR 题名或关键词=双亲))

**Sinomed**

18 results

("父母"[常用字段:智能] OR "父亲"[常用字段:智能] OR "母亲"[常用字段:智能] OR "爸爸"[常用字段:智能] OR "妈妈"[常用字段:智能] OR "双亲"[常用字段:智能]) AND ("健康相关生活质量"[常用字段:智能] OR "健康相关生命质量"[常用字段:智能] OR "健康相关生存质量"[常用字段:智能] OR "生活质量"[常用字段:智能]) AND ("先天性心脏病"[常用字段:智能] OR "先天性心脏缺陷"[常用字段:智能] OR "先天性心脏畸形"[常用字段:智能] OR "先天性心脏血管病"[常用字段:智能])
