# Supplementary material for: Health-related quality of life and influencing factors in parents of children with congenital heart disease: a systematic review and meta-analysis
Source: Front Public Health. 2025 Oct 10;13:1622491. doi: 10.3389/fpubh.2025.1622491 (PMC12550949; doi:10.3389/fpubh.2025.1622491)
Supplement: Supplementary file 2 [file Supplementary_file_2.docx]

### **Critical Appraisal Results**

**Insert text here**

#### **Table 1: Critical Appraisal of Eligible Analytical Cross-Sectional Study**

| **Citation** | **Q1** | **Q2** | **Q3** | **Q4** | **Q5** | **Q6** | **Q7** | **Q8** | **Quality** |
| --- | --- | --- | --- | --- | --- | --- | --- | --- | --- |
| Azhar AS, AlShammasi ZH, Higgi RE. 2016. | Y | Y | Y | Y | Y | Y | Y | Y | High |
| Bevilacqua F, Palatta S, Mirante N, Cuttini M, Seganti G, Dotta A, et al. 2013. | U | Y | Y | Y | U | Y | Y | Y | High |
| Coban N, Ortabag T. 2022. | Y | Y | Y | Y | Y | Y | Y | Y | High |
| Bektas İ, Kır M, Yıldız K, Genç Z, Bektas M, Ünal N. 2020. | Y | Y | Y | Y | Y | Y | Y | Y | High |
| Delaney RK, Thorpe A, Pinto NM, Ozanne EM, Pershing ML, Hansen LM, et al. 2023. | Y | Y | Y | Y | Y | N | Y | N | High |
| Eagleson K, Justo R, Boyle F, Ware R, Johnson S. 2012. | Y | Y | Y | Y | Y | Y | Y | Y | High |
| Denniss DL, Sholler GF, Costa DSJ, Winlaw DS, Kasparian NA. 2019. | Y | Y | Y | Y | Y | Y | Y | Y | High |
| Casey T, Matthews C, Lavelle M, Kenny D, Hevey D. 2024. | Y | Y | Y | Y | Y | Y | Y | Y | High |
| Goldbeck L, Melches J. 2006. | Y | Y | Y | Y | Y | Y | Y | Y | High |
| Golfenshtein N, Lisanti AJ, Medoff-Cooper B. 2023. | Y | Y | Y | Y | Y | Y | Y | Y | High |
| Kaugars A, Shields C, Brosig C. 2018. | Y | Y | Y | Y | Y | Y | Y | Y | High |
| Landolt MA, Buechel EV, Latal B. 2011. | Y | Y | Y | Y | Y | Y | Y | Y | High |
| Lin W-H, Chen Y-K, Lin S-H, Cao H, Chen Q. 2024. | Y | Y | Y | Y | Y | Y | Y | Y | High |
| Liu J-F, Xie W-P, Lei Y-Q, Cao H, Chen Q. 2022. | Y | Y | Y | Y | Y | Y | Y | Y | High |
| Sileshi L, Tefera E. 2017. | N | Y | Y | Y | U | U | Y | Y | High |
| Utens EMWJ, Levert EM, Dulfer K, Van Domburg RT, Helbing WA. 2016. | Y | Y | Y | Y | Y | Y | Y | Y | High |
| Liang Qiuyue, et al 2022. | Y | Y | Y | Y | N | N | Y | Y | High |
| Liao Xinxi, Hu Guimei, Chen Yuchan et al. 2018. | Y | Y | Y | Y | U | U | Y | Y | High |
| Mussatto KA, Trachtenberg FL, Wang K, Uzark K, Sood E, Lambert L, et al. 2023. | Y | Y | Y | Y | Y | Y | Y | Y | High |
| Lee JS, Cinanni N, Di Cristofaro N, Lee S, Dillenburg R, Adamo KB, et al. 2020. | Y | Y | Y | Y | Y | Y | Y | Y | High |
| Lawoko S, Soares JJF. 2003. | Y | Y | Y | Y | U | Y | Y | Y | High |
| Khoshhal S, Al-Harbi K, Al-Mozainy I, Al-Ghamdi S, Aselan A, Allugmani M, et al. 2019. | Y | Y | Y | Y | Y | Y | Y | Y | High |
| % | 90.9 | 100.0 | 100.0 | 100.0 | 77.27 | 81.81 | 100.0 | 95.45 |  |

#### **Table 2: Critical Appraisal of Eligible Analytical Cohort Study**

| Study, year | Selection | Comparability | Outcome | NOS Score |
| --- | --- | --- | --- | --- |
| Alkan F 2017 | 3 | 2 | 2 | 7 |
