# Supplementary material for: Health-related quality of life and influencing factors in parents of children with congenital heart disease: a systematic review and meta-analysis
Source: Front Public Health. 2025 Oct 10;13:1622491. doi: 10.3389/fpubh.2025.1622491 (PMC12550949; doi:10.3389/fpubh.2025.1622491)

**Publication bias**

| **Number** | **study** | **Variable** | **Egger's test** | |
| --- | --- | --- | --- | --- |
|  |  |  | ***t*** | ***P* value** |
| 1 | Lee(2020),  Lawoko(2003),  Khoshhal(2019) | HRQOL | 2.54 | 0.239 |
| 2 | Eagleson(2012),  Denniss(2019),  Kaugars(2018),  Lee(2020) | HRQOL(PedsQL FIM) | -2.98 | 0.097 |
| 3 | Alkan(2017),  Landolt(2011),  Sileshi(2017),  Liao(2018),  Liu(2021) | SF-36(Physical function) | -2.55 | 0.084 |
| 4 | Alkan(2017),  Landolt(2011),  Sileshi(2017),  Liao(2018),  Liu(2021) | SF-36(Social function) | -1.72 | 0.185 |
| 5 | Alkan(2017),  Landolt(2011),  Sileshi(2017),  Liao(2018),  Liu(2021) | SF-36(Role difficulty due to physical problems) | -0.31 | 0.776 |
| 6 | Alkan(2017),  Landolt(2011),  Sileshi(2017),  Liao(2018),  Liu(2021) | SF-36(Role difficulty due to emotional problems) | 2.0 | 0.14 |
| 7 | Alkan(2017),  Landolt(2011),  Sileshi(2017),  Liao(2018),  Liu(2021) | SF-36(Mental health) | -1.04 | 0.374 |
| 8 | Alkan(2017),  Landolt(2011),  Sileshi(2017),  Liao(2018),  Liu(2021) | SF-36(Vitality) | 1.05 | 0.371 |
| 9 | Alkan(2017),  Landolt(2011),  Sileshi(2017),  Liao(2018),  Liu(2021) | SF-36(Pain) | 1.51 | 0.229 |
| 10 | Alkan(2017),  Landolt(2011),  Sileshi(2017),  Liao(2018),  Liu(2021) | SF-36(Vitality) | -0.59 | 0.597 |
| 11 | Khoshhal(2019),  Lin(2024),  Coban(2022) | WHOQOL-BREF(Physical) | 5.03 | 0.125 |
| 12 | Khoshhal(2019),  Lin(2024),  Coban(2022) | WHOQOL-BREF(Psychological) | 0.39 | 0.765 |
| 13 | Khoshhal(2019),  Lin(2024),  Coban(2022) | WHOQOL-BREF(Social) | 1.03 | 0.492 |
| 14 | Khoshhal(2019),  Lin(2024),  Coban(2022) | WHOQOL-BREF(Environmental) | 1.10 | 0.469 |
| 15 | Bevilacqua(2013),  Landolt(2011),  Lawoko(2003),  Azhar(2016) | Gender(Mental health) | -0.50 | 0.669 |
| 16 | Bevilacqua(2013),  Landolt(2011),  Lawoko(2003),  Azhar(2016) | Gender(physical health) | -0.18 | 0.872 |
| 17 | Lin(2024),  Mussatto(2023),  Lawoko(2003) | Gender | 2.69 | 0.227 |

**Sensitivity analysis**

**Number 1**


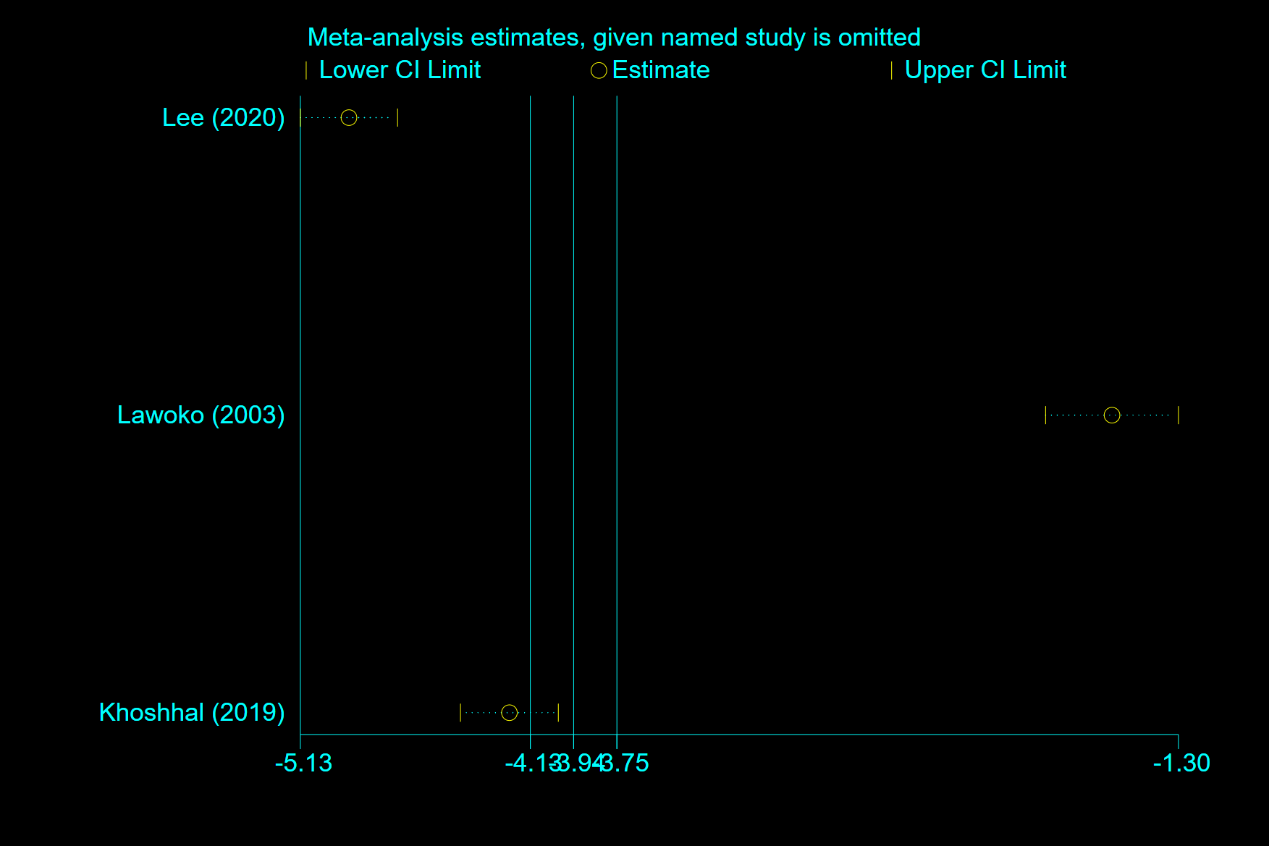


**Number 2**


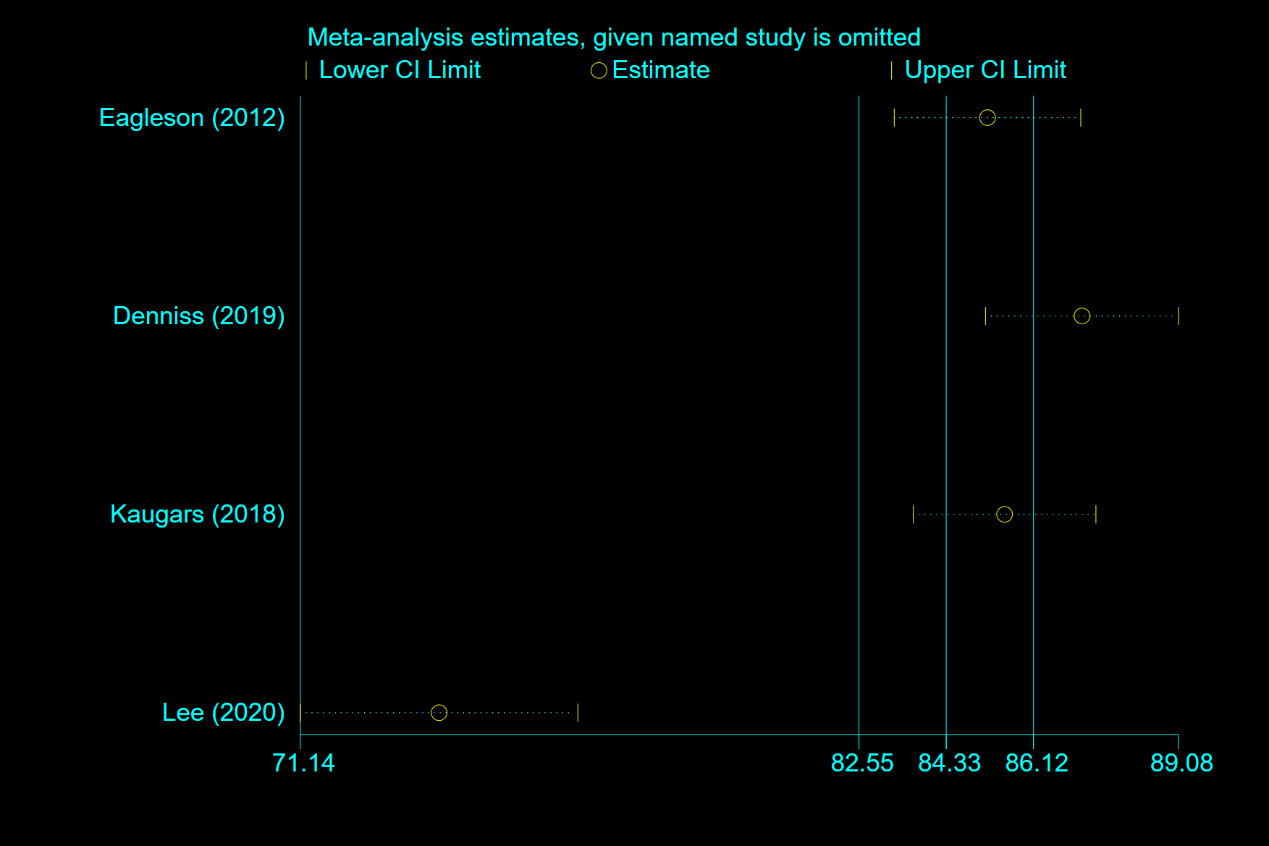


**Number 3**


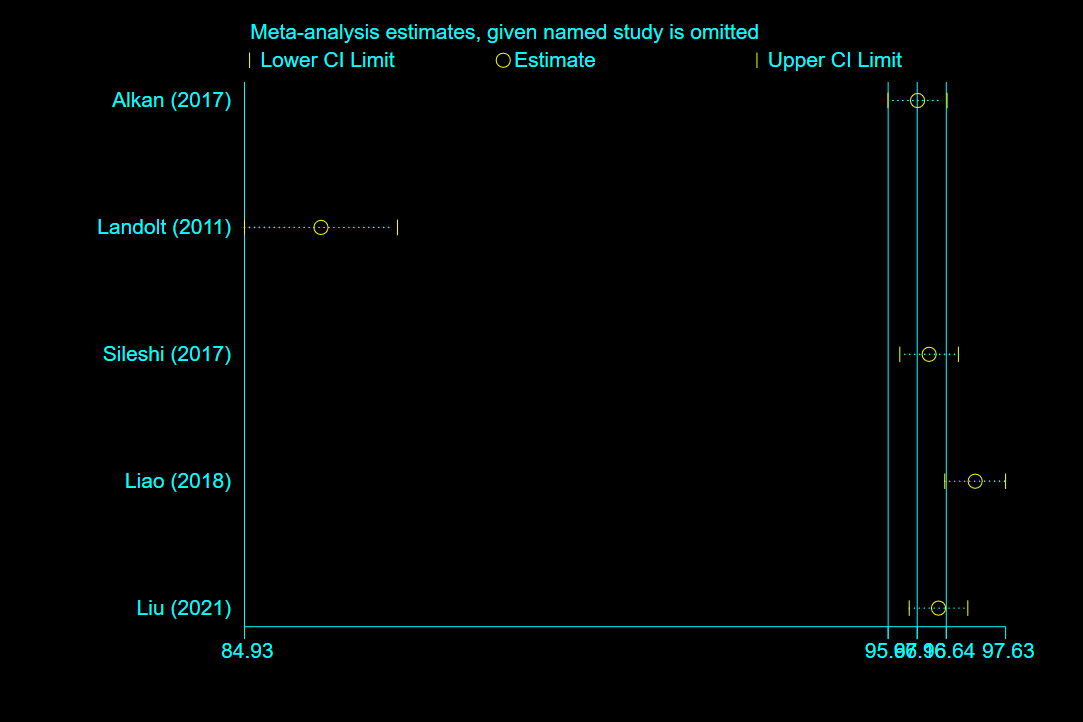


**Number 4**


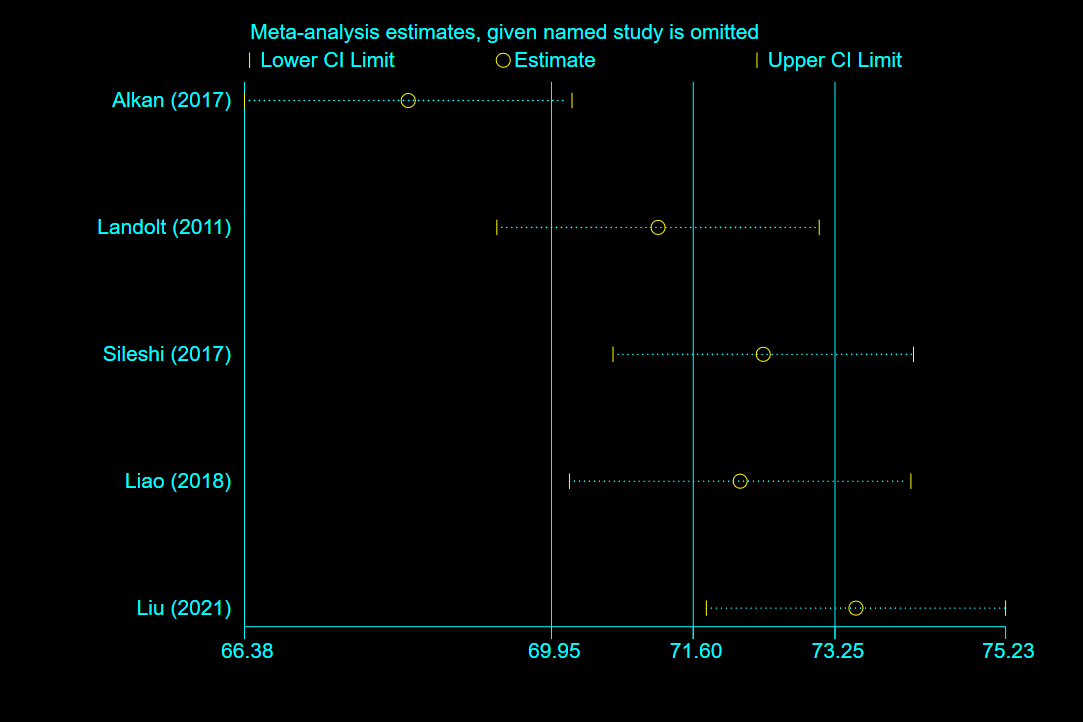


**Number 5**


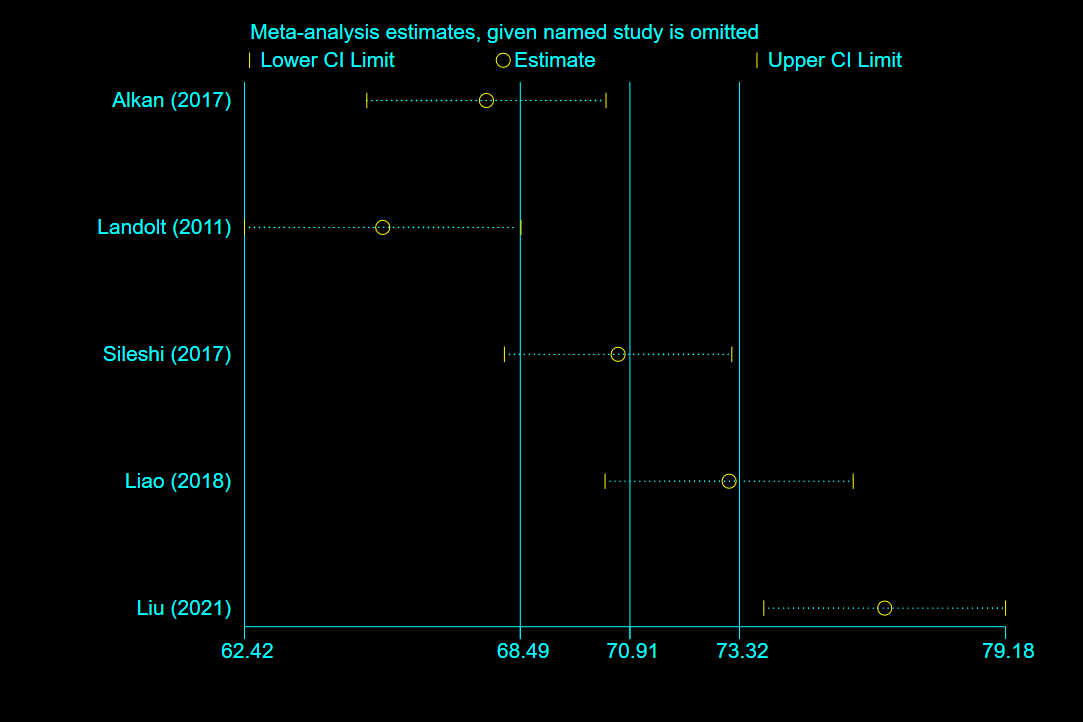


**Number 6**


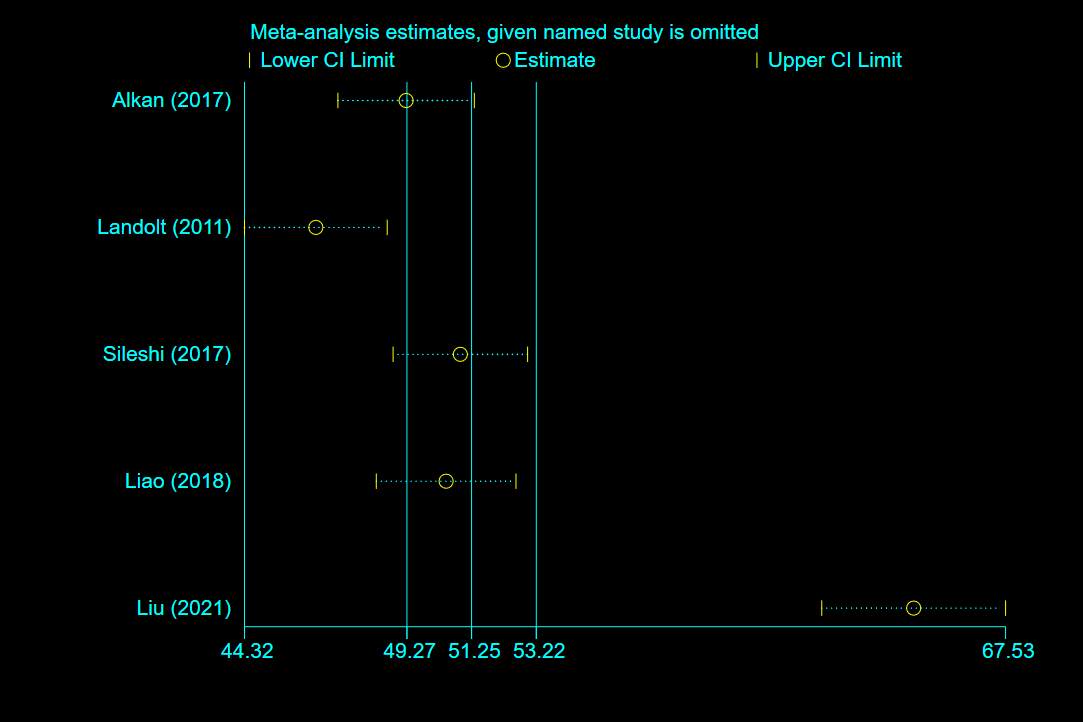


**Number 7**


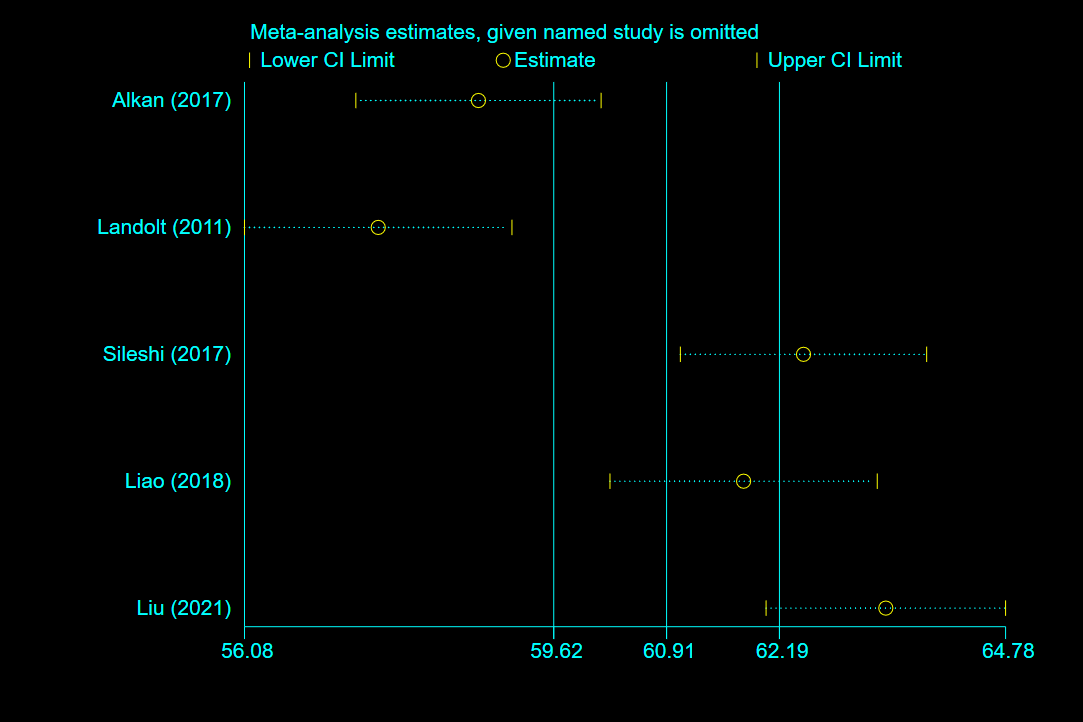


**Number 8**


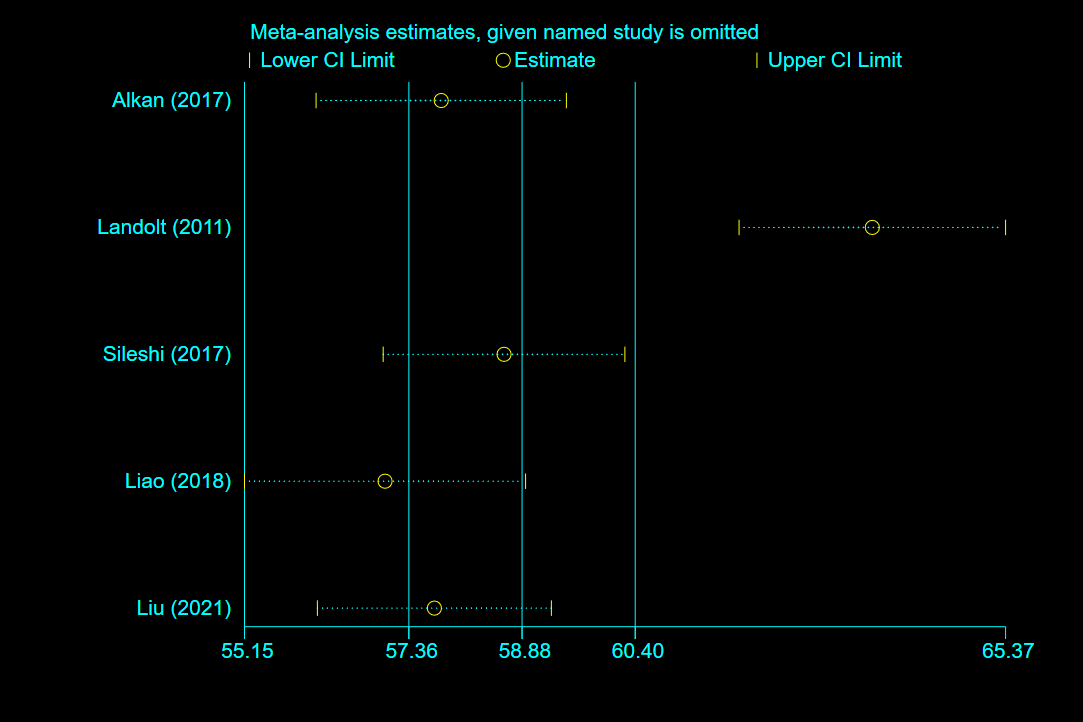


**Number 9**


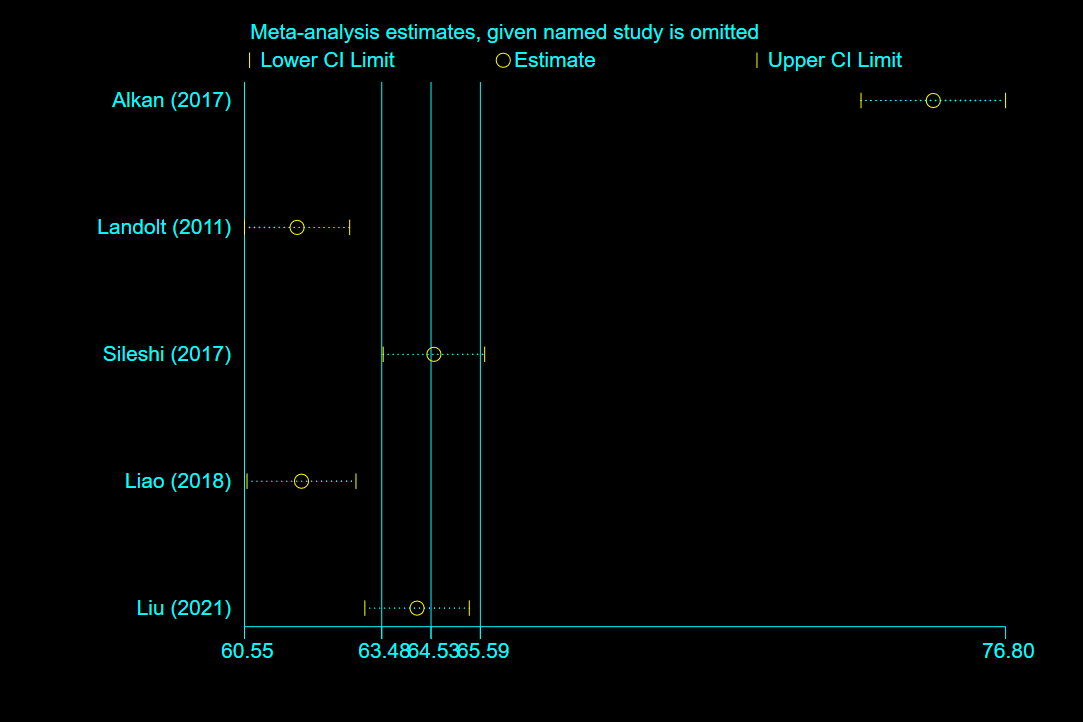


**Number 10**


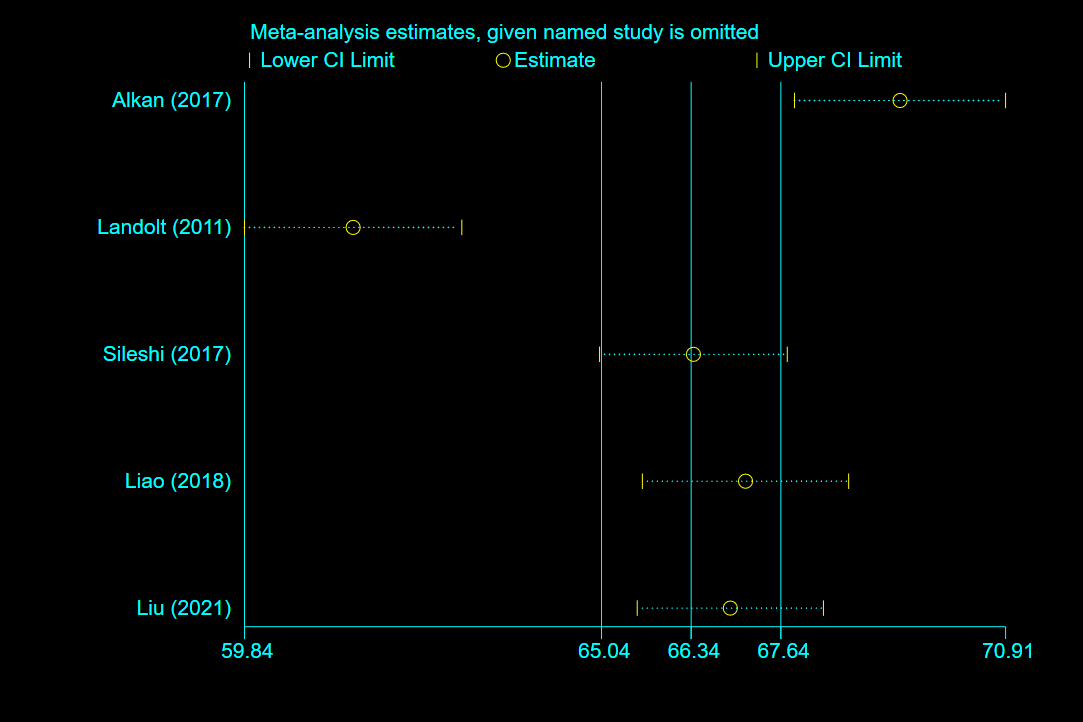


**Number 11**


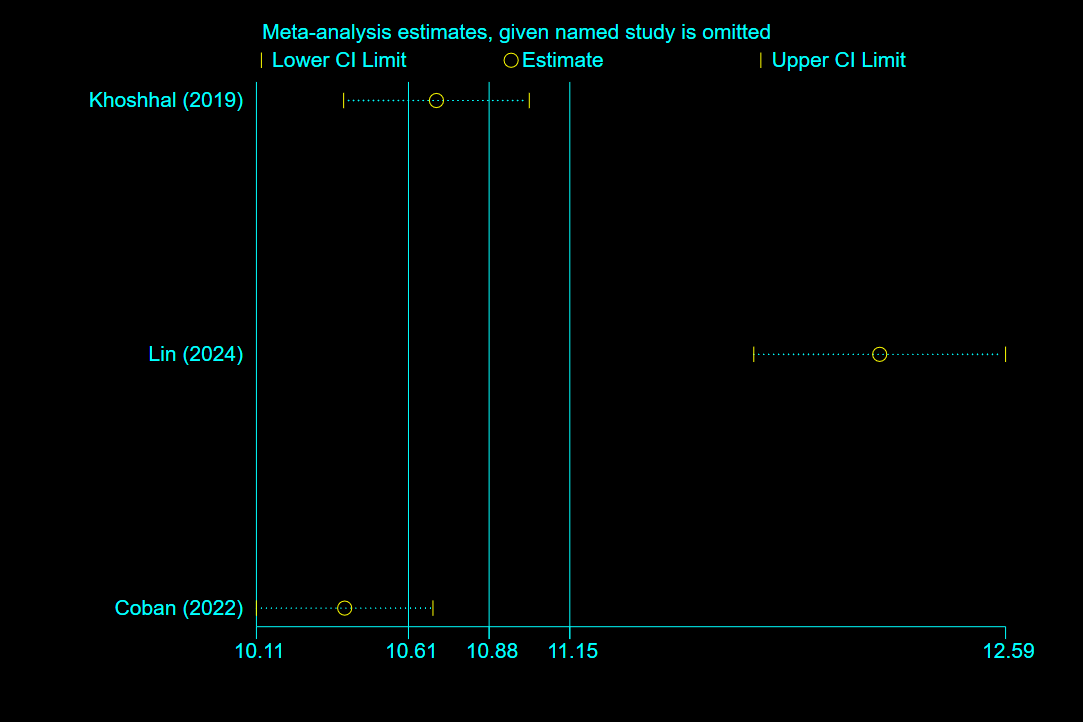


**Number 12**


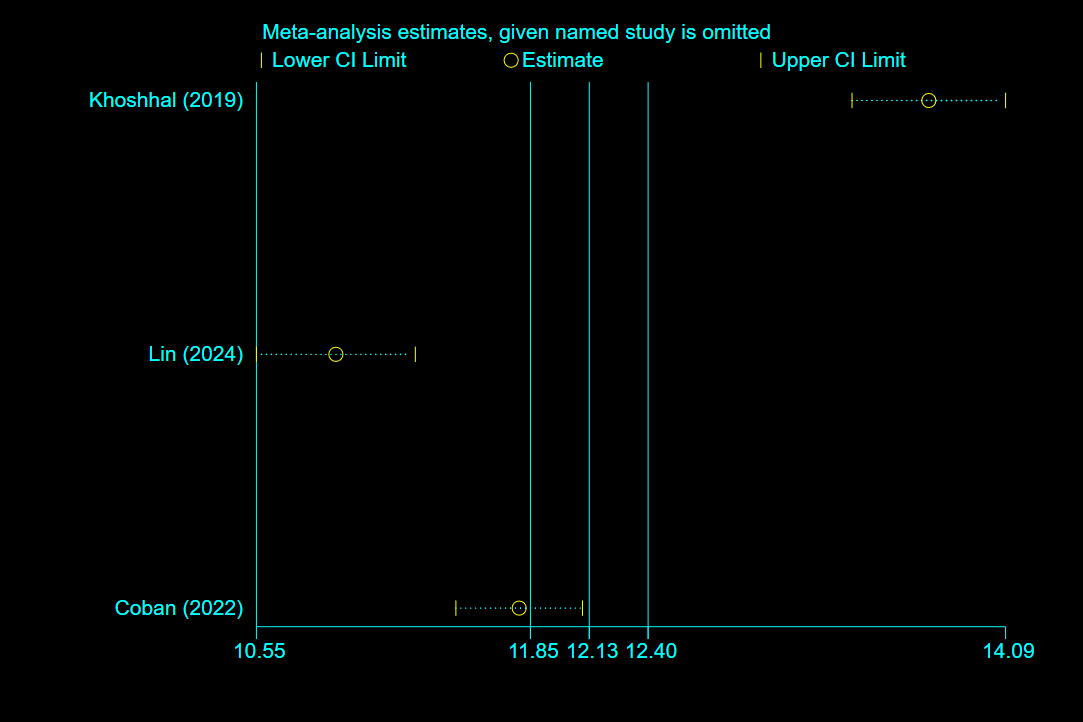


**Number 13**


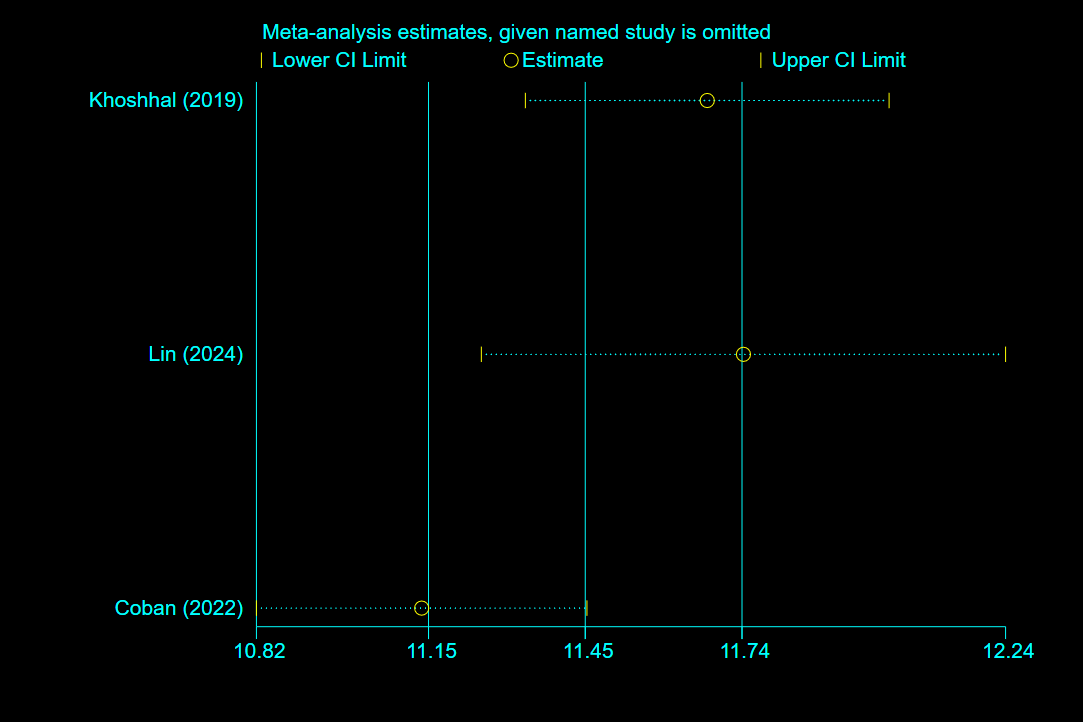


**Number 14**


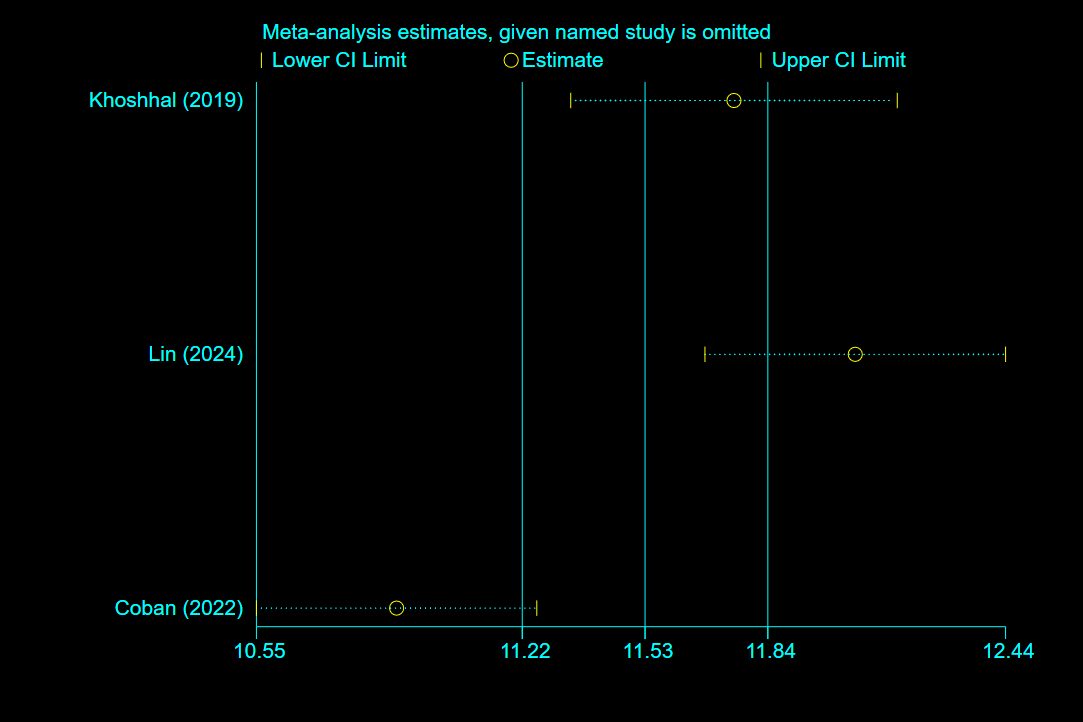


**Number 15**


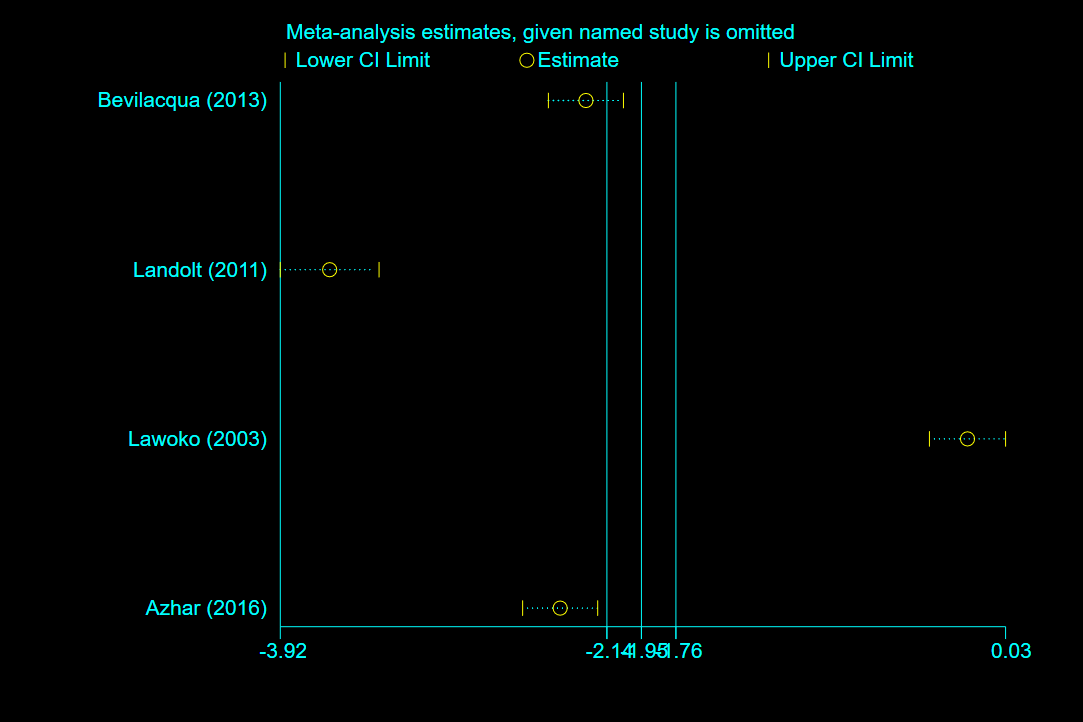


**Number 16**


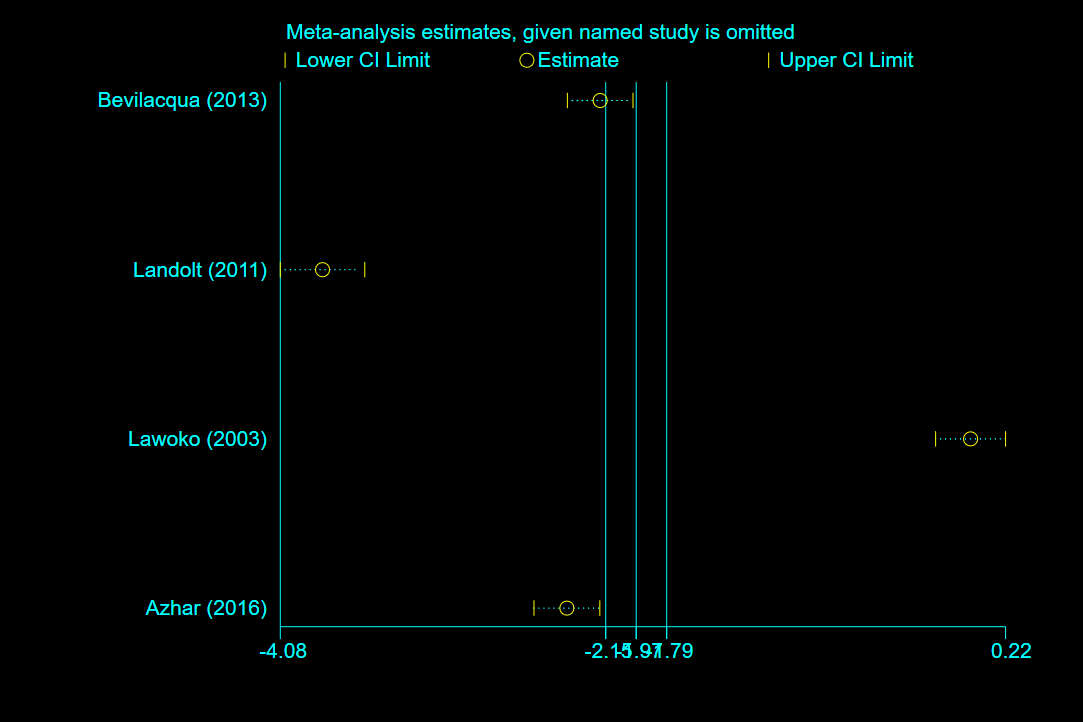


**Number 17**


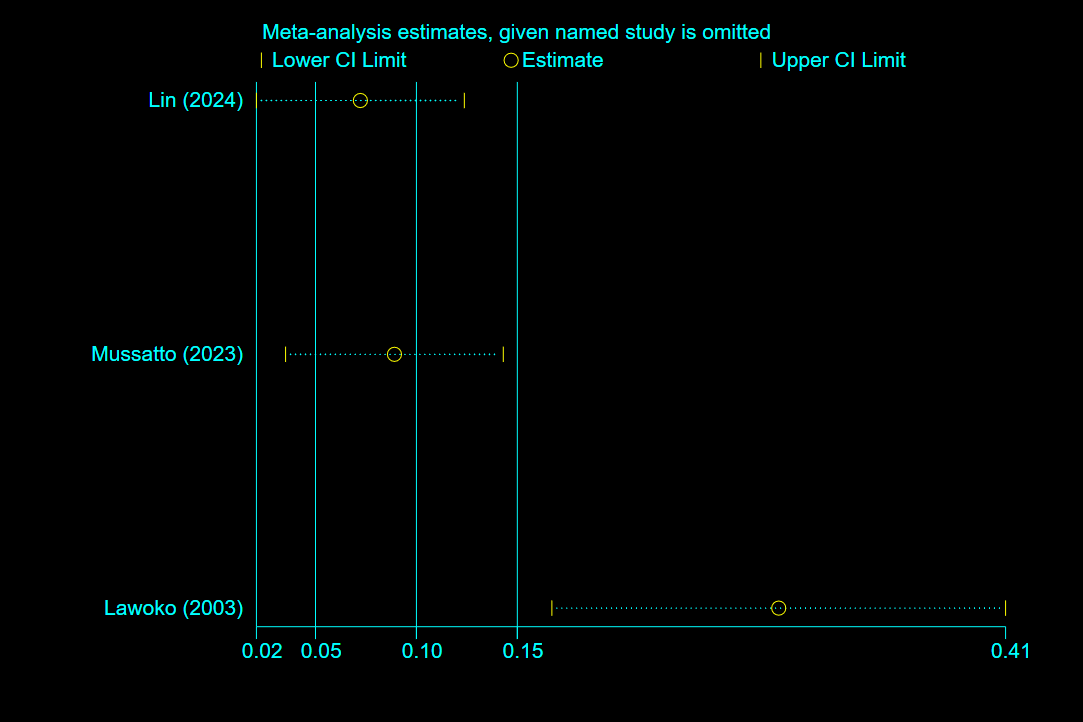

Supplement: Supplementary file 3 [file Supplementary_file_3.zip › Supplementary File 3.docx]
